# Association of Distinct Fine Specificities of Anti−Citrullinated Peptide Antibodies With Elevated Immune Responses to *Prevotella intermedia* in a Subgroup of Patients With Rheumatoid Arthritis and Periodontitis

**DOI:** 10.1002/art.40227

**Published:** 2017-10-30

**Authors:** Anja Schwenzer, Anne‐Marie Quirke, Anna M. Marzeda, Alicia Wong, Anna B. Montgomery, Harlan R. Sayles, Sigrun Eick, Katarzyna Gawron, Maria Chomyszyn‐Gajewska, Katarzyna Łazarz‐Bartyzel, Simon Davis, Jan Potempa, Benedikt M. Kessler, Roman Fischer, Patrick J. Venables, Jeffrey B. Payne, Ted R. Mikuls, Kim S. Midwood

**Affiliations:** ^1^ University of Oxford Oxford UK; ^2^ University of Oxford, Oxford, UK, and Jagiellonian University Krakow Poland; ^3^ Jagiellonian University Krakow Poland; ^4^ University of Nebraska Medical Center Omaha; ^5^ University of Bern Bern Switzerland; ^6^ Jagiellonian University, Krakow, Poland, and University of Louisville Louisville Kentucky; ^7^ University of Nebraska Medical Center Lincoln; ^8^ University of Nebraska Medical Center and Nebraska‐Western Iowa Health Care System Omaha

## Abstract

**Objective:**

In addition to the long‐established link with smoking, periodontitis (PD) is a risk factor for rheumatoid arthritis (RA). This study was undertaken to elucidate the mechanism by which PD could induce antibodies to citrullinated peptides (ACPAs), by examining the antibody response to a novel citrullinated peptide of cytokeratin 13 (CK‐13) identified in gingival crevicular fluid (GCF), and comparing the response to 4 other citrullinated peptides in patients with RA who were well‐characterized for PD and smoking.

**Methods:**

The citrullinomes of GCF and periodontal tissue from patients with PD were mapped by mass spectrometry. ACPAs of CK13 (cCK13), tenascin‐C (cTNC5), vimentin (cVIM), α‐enolase (CEP‐1), and fibrinogen β (cFIBβ) were examined by enzyme‐linked immunosorbent assay in patients with RA (n = 287) and patients with osteoarthritis (n = 330), and cross‐reactivity was assessed by inhibition assays.

**Results:**

A novel citrullinated peptide cCK13‐1 (_444_ TSNASGR‐Cit‐TSDV‐Cit‐RP_458_) identified in GCF exhibited elevated antibody responses in RA patients (24%). Anti–cCK13‐1 antibody levels correlated with anti–cTNC5 antibody levels, and absorption experiments confirmed this was not due to cross‐reactivity. Only anti–cCK13‐1 and anti‐cTNC5 were associated with antibodies to the periodontal pathogen *Prevotella intermedia* (*P* = 0.05 and *P* = 0.001, respectively), but not with antibodies to *Porphyromonas gingivalis* arginine gingipains. Levels of antibodies to CEP‐1, cFIBβ, and cVIM correlated with each other, and with smoking and shared epitope risk factors in RA.

**Conclusion:**

This study identifies 2 groups of ACPA fine specificities associated with different RA risk factors. One is predominantly linked to smoking and shared epitope, and the other links anti–cTNC5 and cCK13‐1 to infection with the periodontal pathogen *P intermedia*.

Rheumatoid arthritis (RA) is an autoimmune disease characterized by the generation of disease‐specific autoantibodies against modified protein antigens 1, 2, 3. The best‐studied autoantibodies in RA to date are those that recognize citrullinated peptides, whose epitopes are created by the posttranslational conversion of arginine residues to citrulline residues by peptidylarginine deiminases (PADs). Among an increasing number of citrullinated proteins identified as antigens for autoantibodies in RA, peptides of citrullinated fibrinogen β (cFIBβ) 4, citrullinated vimentin (cVIM) 5, citrullinated α‐enolase peptide 1 (CEP‐1) 6, and citrullinated tenascin‐C (cTNC5) 7 are well characterized, and have been confirmed as diagnostically sensitive and specific in independent cohorts.

Despite the fact that anti–citrullinated peptide antibodies (ACPAs) are excellent biomarkers for RA and widely used in disease diagnosis 8, it is still not clear what triggers their production and how this breach of tolerance occurs, nor is the contribution of these autoantibodies to disease pathogenesis completely understood 9. The fact that ACPAs can be detected years before any overt clinical symptoms of RA 10, together with the association of RA with risk factors including smoking 11 and periodontitis (PD) 12, raises the possibility that these antibodies arise due to events outside the joints, for example in the lungs or periodontium.

PD is a chronic inflammatory disease of the periodontium, characterized by the destruction of both soft and hard tissue, and, ultimately, tooth loss. It is associated with pathogen invasion of periodontal pockets, creating a shift in the oral microbiota from a symbiotic to a dysbiotic community. Among these dysbiotic species, an increased frequency of *Porphyromonas gingivalis,* a key PD‐associated pathogen, is accompanied by elevated levels of many other diverse species that are also linked to PD, including *Prevotella intermedia* and *Fusobacterium nucleatum* 13.

Citrullination has been observed in gingival tissue from patients with PD by staining with pan–anticitrulline‐specific antibodies 14, 15, and citrulline levels are elevated in gingival crevicular fluid (GCF) samples from PD patients 16. In a recent study, Konig et al 17 demonstrated by mass spectrometry that a wide range of proteins were citrullinated in GCF from patients with PD, compared to minimal citrullination in subjects without PD. However, that study did not examine the antibody response to citrullinated GCF proteins, and whether this could be related to PD in patients with RA. Citrullination in PD may be mediated by endogenous human PAD or by a bacterial PAD (PPAD) specifically expressed by *P gingivalis*. PPAD preferentially citrullinates C‐terminal arginines exposed by the action of the bacterial gingipain proteases (R gingipain type A [RgpA] and RgpB) that cleave both bacterial and host proteins in PD tissue 18, 19. Taken together, these data suggest a causal link between RA and PD, wherein citrullinated proteins generated in the periodontium may provide a primary source of autoantibody epitopes that play a role in the initiation of RA 20.

To further investigate the possibility that citrullination of proteins in PD contributes to the autoantibody response in RA, we analyzed GCF and periodontal tissues from patients with PD by mass spectrometry. Within the periodontal citrullinome, we found citrullinated proteins previously known to be targets of ACPAs in RA. We also detected novel citrullinated sites, among which was a peptide of cytokeratin 13 (cCK13‐1). By comparing antibody responses to this novel peptide and 4 other well‐established ACPA antigens, we identified distinct patient subsets, each with different links to different RA risk factors.

## Patients and Methods

**Subjects.** GCF was obtained from a total of 8 study participants, including 2 controls without RA or PD, 3 patients with both RA and PD, 2 patients with PD but not RA, and 1 patient with RA but not PD 16. Multiple periodontal tissue samples were collected from a single PD patient undergoing dental extraction, with informed consent and ethical approval. Tissue samples were washed with sterile phosphate buffered saline immediately after collection and snap‐frozen. No tissue was available from healthy subjects because of ethical considerations.

The cohort used to analyze the antibody response to citrullinated peptides has been described in detail previously 12. This cohort comprises serum from 617 study participants, including 287 RA patients (100 patients with PD) and 330 osteoarthritis (OA) patients (87 patients with PD). RA patients in this study had established disease with a mean disease duration exceeding 10 years. A vast majority had received disease‐modifying therapies including, but not limited to, methotrexate (62%), glucocorticoids (30%), and/or biologic agents (31%). Serum samples from a separate group of RA patients were used to analyze cross‐reactivity 6. All RA cases fulfilled the American College of Rheumatology 1987 classification criteria 21. PD was defined a priori according to the definition of Machtei et al as the presence of clinical attachment loss ≥6 mm on ≥2 teeth and ≥1 sites with probing depths ≥5 mm 22. This definition equates closely to the US Centers for Disease Control and Prevention/American Academy of Periodontology case definition for “severe” PD 23, 24. Of those classified as having PD, approximately one‐fourth reported having prior periodontal treatment and 16% reported prior periodontal surgery, with frequencies that were similar in cases and controls 25.

Genotyping for the HLA–DRB1 shared epitope and enzyme‐linked immunosorbent assays (ELISAs) to measure IgG antibody responses to outer membrane antigens of *P gingivalis, P intermedia,* and *F nucleatum* are described in detail in the study by Mikuls et al 12. Nested polymerase chain reaction (PCR) was used for the detection of *P gingivalis* in subgingival plaque samples 26.

**Liquid chromatography mass spectrometry/mass spectrometry (LC‐MS/MS) analyses of GCF and PD tissue.** GCF and PD tissue samples were stored at −80°C. GCF was prepared for LC‐MS/MS analysis as described previously 27. Briefly, GCF was reduced and alkylated with dithiothreitol/iodoacetamide, followed by protein precipitation using chloroform and methanol. The protein pellet was solubilized in 6*M* urea, diluted to 1*M* urea with ammonium bicarbonate, digested with trypsin, and desalted on reverse‐phase material (Sola C18; Thermo Fisher).

Inflammatory gingival tissue surrounding the tooth and subgingival plaque from root planing of the extracted tooth was first lysed using Precellys bead beating (Bertin Instruments) in radioimmunoprecipitation assay buffer (6,500 revolutions per minute for 40 seconds, followed by resting for 5 minutes on ice, repeated 3 times), followed by centrifugation at 15,000 rpm in the cold. The supernatant was prepared for LC‐MS/MS analysis as described above.

GCF and gingival tissue samples were analyzed on an LC‐MS/MS workflow comprising a Dionex Ultimate 3000 nLC system coupled to a Q‐Exactive mass spectrometer (Thermo Scientific) 28. Briefly, chromatographic separation was achieved using a 50 cm nEASY spray column (PepMAP C18; 75 μm × 500 mm, 2 μm particle size) (Thermo Scientific) and a linear acetonitrile gradient from 2−35% in 5% DMSO and 0.1% formic acid. Precursor peptides were detected with a resolution of 70,000 at 200 mass/charge followed by the selection of up to 15 precursor ions. Raw data were imported into Progenesis QI V4.1.4832.42146 (Waters) for label‐free quantitation and alignment, and peptides were identified with PEAKS, version 7 (Bioinformatics Solutions).

**Validation of citrullinated peptides.** MS/MS spectra were validated manually. We confirmed whether retention times were altered if a noncitrullinated version of the peptide was detected. We also made sure that the precursor selection assigned the correct precursor mass and not the ^13^C peak. This was repeated for all assigned fragment ions in the corresponding MS/MS spectra as well. In addition, we shortlisted citrullinated peptides with missed cleavage sites in the tryptic digests. Deamidation was excluded by the assignment of site‐specific fragment ions.

We identified 756 protein groups in PD tissue sample 1 with 4,748 peptide sequences (peptide and protein false discovery rate [FDR] 1%). In PD tissue sample 2 we found 242 protein groups with 2,476 sequences (FDR 1% for peptides and 1.5% for proteins). GCF analysis resulted in the identification of a total of 387 protein groups (FDR 1%) with 3,798 peptide sequences (FDR 1%).

**Peptides and protein.** Sera were tested for antibodies to immunodominant peptides of cTNC5 7, cVIM 5, cFIBβ 29, and CEP‐1 6 as well as antibodies to peptides of cCK13 (accession number NP_002265.2). Peptides were synthesized at a purity of >90%, with C‐terminal and N‐terminal cysteines to facilitate cyclization (Pepceuticals) and solubilized in water at 10 mg/ml. RgpB‐6xHis was purified as previously described 30.

**ELISAs for citrullinated peptides and RgpB.** ACPAs were measured using a second‐generation anti–cyclic citrullinated peptide 2 (anti–CCP‐2) IgG ELISA (Diastat; Axis‐Shield). ELISAs were used to detect antibodies against citrullinated peptides in human sera as previously described 6. Briefly, 96‐well plates were coated with 10 μg/ml peptide (or 5 μg/ml RgpB) 31, blocked with 2% bovine serum albumin, and incubated with sera diluted 1:100. Bound antibodies were detected with a horseradish peroxidase–conjugated anti‐human IgGFc monoclonal antibody (Stratech). A standard curve of positive sera was used to calculate relative antibody titers in arbitrary units for each sample for each peptide. Anti‐RgpB levels were expressed as optical density since the assay was carried out without a standard curve; however, a positive control on each plate gave a coefficient of variance of <15% between plates. Inhibition ELISAs to analyze cross‐reactivity between ACPAs and citrullinated peptides were performed as described previously 7.

**Statistical analysis.** CCP‐2 serum positivity was defined as a level of ≥5 units/ml. The 98th percentile in OA control samples was used to define the cutoff for positive antibody levels. Correlations between antibody levels were calculated using Spearman's rank correlation.

Chi‐square tests were used to compare frequency distributions of categorical variables while *t*‐tests and Wilcoxon's rank sum tests were used to compare all continuous variables. Fisher's exact tests were used in place of chi‐square tests for the CCP‐2 analysis because of the small number of seronegative cases. Venn analysis was conducted using the Venn Diagram Generator (http://www.pangloss.com/seidel/Protocols/venn.cgi) followed by proportional visualization with Venn Diagram Plotter (by Kyle Littlefield and Matthew Monroe, PNNL, Richland, WA).

## Results

**Citrullinated proteins in GCF and periodontal tissue.** We identified 24 citrullinated peptides of 8 proteins in GCF (Supplementary Table 1, available on the *Arthritis & Rheumatology* web site at http://onlinelibrary.wiley.com/doi/10.1002/art.40227/abstract) and 92 citrullinated peptides of 56 proteins in PD tissue (Supplementary Table 2, available on the *Arthritis & Rheumatology* web site at http://onlinelibrary.wiley.com/doi/10.1002/art.40227/abstract). In GCF, citrullinated peptides were detected in samples from patients with both PD and RA, but also in samples from patients with RA or PD alone, and in samples from healthy controls. A number of the citrullinated sites that we identified in this study have previously been identified in RA synovial tissue or fluid, and demonstrated to act as autoantigen epitopes, including R35 in fibrinogen α 32, R715 in α_2_‐macroglobulin, R450 and R304 in vimentin, R93 in histone H2B type 2‐E 33, R4 in histone 2A 34, as well as R69 and R71 in vimentin 29. We also detected citrullinated peptides in GCF and periodontal tissues that have not been described as autoantigens in RA. Noteworthy were a number of peptides of CK‐13, which were detected in 7 of the 8 GCF samples examined, but not in the tissue sample (Table 1 and Supplementary Figure 1, available on the *Arthritis & Rheumatology* web site at http://onlinelibrary.wiley.com/doi/10.1002/art.40227/abstract). These data demonstrate that citrullinated peptides are present in GCF and in periodontal tissue from a patient with PD.

**Table 1:** Citrullinated CK‐13 peptides identified in GCF from healthy controls, patients with both PD and RA, patients with PD only, and patients with RA only^a^

Citrullinated CK‐13 peptide	Control (n = 2)	PD and RA (n = 3)	PD (n = 2)	RA (n = 1)
_5_LQSSSASYGGGFGGGSCQLGGG(cit)GVSTCSTR_35_ b	1	0	0	0
_326_RTLQGLEIELQSQLSMKAGLENTVAETEC(cit)_355_ c	1	0	0	0
_407_SLLEGQDAKMIGFPSSAGSVSP(cit)R_430_ b	1	0	0	0
_430_STSVTTTSSASVTTTSNASG(cit)R_451_ b, c	1	1	2	0
_451_(cit)TSDV(cit)RP_458_ b, c	1	2	1	0
_451_(cit)TSDVR(cit)P_458_ b, c	1	2	1	0
_452_TSDV(cit)RP_458_ c	1	1	1	1
_452_TSDVR(cit)P_458_ c	1	1	0	0

a Values are the number of patients. CK‐13 = cytokeratin 13; PD = periodontitis; RA = rheumatoid arthritis.

b Gingival crevicular fluid (GCF) with no tryptic digest.

c Tryptic digest of GCF solution.

**Peptide cCK13‐1 is a novel peptide recognized by ACPAs in patients with RA.** CK‐13 expression is restricted to mucosal membranes. This specific localization, combined with the high number of citrullinated peptides identified in the C‐terminal region of this protein, and the enrichment of these peptides in patients with PD (Table 1 and Supplementary Figure 1), indicated this as a good candidate for further examination. We synthesized 2 CK‐13 peptides with citrullines at positions 451 and 456 (cCK13‐1: _444_TSNASGR‐cit‐TSDV‐cit‐RP_458_), or at positions 451 and 457 (cCK13‐2: _444_TSNASGR‐cit‐TSDVR‐cit‐P_458_), and mapped antibody responses to these peptides in a US cohort of 287 RA patients and 330 OA controls. Antibodies to cCK13‐1 had a diagnostic sensitivity of 24% and specificity of 98% in RA, while no antibodies were detected against cCK13‐2 (Table 2) or against arginine control peptides (data not shown). Taken together, these data indicate that an antibody response in RA exists toward a citrullinated epitope that can be generated in inflamed periodontal tissue.

**Table 2:** Frequency of ACPA positivity in 287 RA patients^a^

Antigen	Peptide sequence	Percent of patients
cCK13‐1	_444_TSNASGR‐cit‐TSDV‐cit‐RP_458_	24.0
cCK13‐2	_444_TSNASGR‐cit‐TSDVR‐cit‐P_458_	0
cFIBβ	_36_NEEGFFSA‐cit‐GHRPLDKK_52_	66.2
cTNC5	_2176_EHSIQFAEMKL‐cit‐PSNF‐cit‐NLEG‐cit‐cit‐KR_2200_	50.5
CEP‐1	_5_KIHA‐cit‐EIFDS‐cit‐GNPTVE_21_	39.4
cVIM	_59_VYAT‐cit‐SSAV‐cit‐L‐cit‐SSVP_74_	32.1
CCP‐2	–	84.5

a Antibody sensitivity was 98% for all protein antigens. ACPA = anti–citrullinated peptide antibody; RA = rheumatoid arthritis; cCK13‐1 = citrullinated cytokeratin 13–derived peptide 1; cFIBβ = citrullinated fibrinogen β; cTNC5 = citrullinated tenascin‐C; CEP‐1 = citrullinated α‐enolase peptide 1; cVIM = citrullinated vimentin; CCP‐2 = cyclic citrullinated peptide 2.

**Figure 1:**
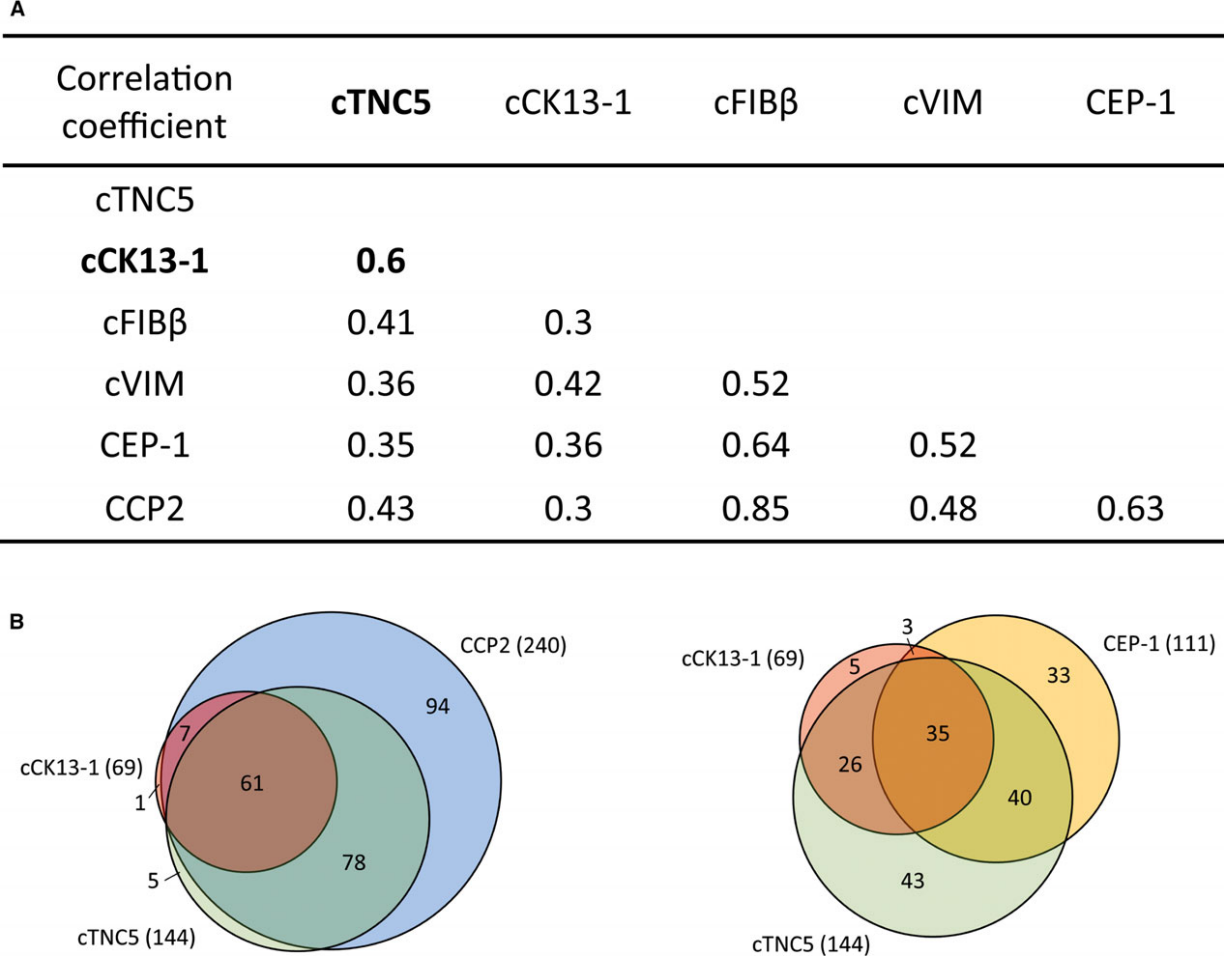
**A,** Spearman's rank correlation coefficients for the correlations between different anti−citrullinated peptide antibodies (ACPAs) in rheumatoid arthritis (RA) patients. *P* < 0.001 for all correlations. Boldface indicates the correlation between citrullinated cytokeratin 13 (cCK13‐1) and citrullinated tenascin‐C (cTNC5) antibody levels. **B,** Venn diagrams showing the overlap between cTNC5, cCK13‐1, and cyclic citrullinated peptide 2 (CCP‐2) antibody responses (left) and between cTNC5, cCK13‐1, and citrullinated α‐enolase peptide 1 (CEP‐1) responses (right). Values in the diagrams are the number of patients in each subset, and values in parentheses are the total number of ACPA‐positive patients. cFIBβ = citrullinated fibrinogen β; cVIM = citrullinated vimentin.

**Correlation of anti−cCK13‐1 antibody levels with anti‐cTNC5 antibody levels.** In the same cohort, antibodies to CEP‐1, cFIBβ, cVIM, cTNC5, and CCP‐2 were also elevated, with cFIBβ having the best diagnostic sensitivity (66%) of the ACPA fine specificities, and CCP‐2 exhibiting the highest levels overall (85%) (Table 2). Anti−cCK13‐1 antibody levels correlated most strongly with anti‐cTNC5 antibody levels (r = 0.60, *P* < 0.001) and only moderately with other antibody levels (r = 0.30 for cFIBβ, r = 0.42 for cVIM, r = 0.36 for CEP‐1, and r = 0.30 for CCP‐2; all *P* < 0.001) (Figure 1A). There was a strong overlap between cTNC5 and cCK13‐1 positivity, as 88% of patients who were positive for cCK13‐1 were also positive for cTNC5. There was only a partial overlap between patients who were positive for cCK13‐1 and those who were positive for CEP‐1, as only 55% of patients who were positive for cCK13‐1 were also CEP‐1 positive (Figure 1B).

**Figure 2:**
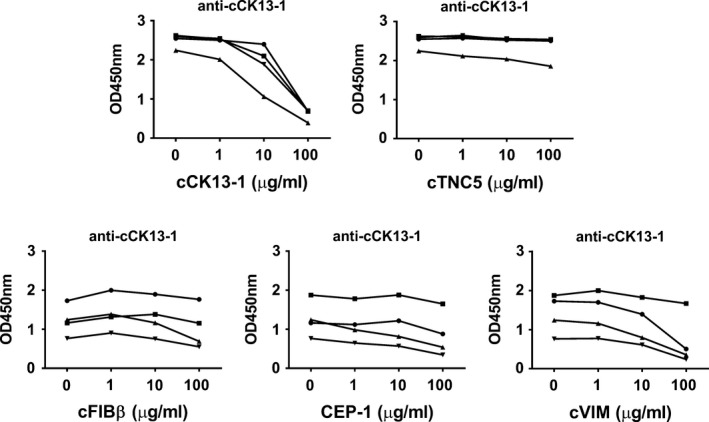
Anti–citrullinated cytokeratin 13 (anti–cCK13‐1) antibody cross‐reactivity with citrullinated tenascin‐C (cTNC5), citrullinated fibrinogen β (cFIBβ), citrullinated α‐enolase peptide 1 (CEP‐1), and citrullinated vimentin (cVIM). Serum from patients positive for both anti–cCK13‐1 and the test antibodies were preincubated with increasing concentrations of the indicated test peptide, and the IgG responses to cCK13‐1 were measured by enzyme‐linked immunosorbent assay and presented as the OD. Each symbol represents an individual patient.

To examine epitope specificity and potential cross‐reactivity of anti–cCK13‐1 antibodies with other antigens targeted by ACPAs, inhibition experiments were performed with CEP‐1, cFIBβ, cTNC5, and cVIM. Adsorption by the homologous peptide was efficient for cCK13‐1. There was no cross‐reactivity between anti−cCK13‐1 and cTNC5, and only limited cross‐reactivity between anti–cCK13‐1 and cFIBβ (1 of 4 sera exhibiting cross‐reactivity). There was some cross‐reactivity between anti–cCK13‐1 and CEP‐1 (2 of 4 sera exhibiting inhibition levels up to 57%) and with anti‐cVIM in particular, with 3 of 4 sera exhibiting inhibition levels >65% (Figure 2). However, cCK13‐1 showed no sequence homology with either peptide (Table 2).

**Association of ACPAs with RA risk factors and with PD pathogens.** We analyzed how cCK13‐1 antibody positivity associated with the RA risk factors smoking, shared epitope, and PD, in comparison to other established ACPA fine specificities. Specific ACPAs were associated with smoking (cFIBβ [*P* = 0.001] and CEP‐1 [*P* = 0.034]) and the shared epitope (cVIM [*P* < 0.001] and CEP‐1 [*P* = 0.001]) (Table 3). Anti‐cFIBβ antibodies were associated with PD (Table 3), and CCP‐2, CEP‐1, and cFIBβ antibody levels were significantly elevated in RA patients with PD compared to RA patients without PD (Supplementary Figure 2).

**Table 3:** Association of antibodies with risk factors for RA^a^

Risk factor	Smoking history			HLA−DRB1 SE positive	PD
	Never smoked	Former smoker	Current smoker		
CCP‐2+ (n = 240)	37	43	21	81	37
CCP‐2− (n = 44)	45	48	7	49	23
*P*	–	–	0.076	<0.001^b^	0.085^b^
cFIBβ+ (n = 190)	35	39	25	79	41
cFIBβ− (n = 97)	42	51	7	69	24
*P*	–	–	0.001	0.061	0.005
cVIM+ (n = 92)	39	41	20	92	36
cVIM− (n = 195)	37	44	19	68	34
*P*	–	–	0.902	<0.001	0.802
CEP‐1+ (n = 95)	27	48	24	88	41
CEP‐1− (n = 192)	43	41	17	69	32
*P*	–	–	0.034	0.001	0.12
cTNC5+ (n = 145)	35	43	22	80	37
cTNC5− (n = 142)	40	44	16	71	33
*P*	–	–	0.412	0.085	0.539
cCK13‐1+ (n = 69)	42	35	23	72	38
cCK13‐1− (n = 218)	36	46	18	77	34
*P*	–	–	0.256	0.451	0.57

a Values are the percent of patients. Except where indicated otherwise, chi‐square tests were used to compare frequency distributions of categorical variables. RA = rheumatoid arthritis; SE = shared epitope; PD = periodontitis; CCP‐2 = cyclic citrullinated peptide 2; cFIBβ = citrullinated fibrinogen β; cVIM = citrullinated vimentin; CEP‐1 = citrullinated α‐enolase peptide 1; cTNC5 = citrullinated tenascin‐C; cCK13‐1 = citrullinated cytokeratin 13–derived peptide 1.

b By Fisher's exact test.

We also determined the association of ACPA fine specificities with infection with periodontal pathogens. CEP‐1–positive patients and cTNC5‐positive patients had higher serum levels of *P gingivalis* outer membrane antibody compared to antibody‐negative patients (*P* = 0.022 for CEP‐1 and *P* = 0.015 for cTNC5), but no other ACPAs were associated with *P gingivalis* outer membrane antibody. None of the ACPA specificities were associated with *P gingivalis* DNA detected by PCR in subgingival plaques. Patients who were positive for cTNC5 and those who were positive for cCK13‐1 had significantly higher levels of anti−*P intermedia* antibodies (*P* = 0.001 for cTNC5 and *P* = 0.05 for cCK13‐1) (Table 4). This association was confirmed by statistically significant quantitative correlations of both anti‐cTNC5 and anti−cCK13‐1 levels with levels of anti−*P intermedia* antibodies (r = 0.207, *P* = 0.0004 for cTNC5 in all RA patients; r = 0.223, *P* = 0.0005 for cTNC5 in CCP+ RA patients; r = 0.152, *P* = 0.010 for cCK13‐1 in all RA patients; and r = 0.161, *P* = 0.012 for cCK13‐1 in CCP+ RA patients).

**Table 4:** Periodontal pathogen status based on antibody positivity^a^

Periodontal pathogen	*Pg* positive by PCR, % of patients	Anti‐*Pg* outer membrane antibody levels, mean ± SD μg/ml	Anti‐RgpB at OD450, mean ± SD μg/ml	Anti‐*Pi* outer membrane antibody levels, mean ± SD μg/ml	Anti‐*Fn* outer membrane antibody levels, mean ± SD μg/ml
CCP‐2+ (n = 240)	61	106 ± 90	0.3 ± 0.4	145 ± 76	111 ± 115
CCP‐2− (n = 44)	61	96 ± 84	0.3 ± 0.5	146 ± 93	96 ± 74
*P*	1.000^b^	0.365^c^	0.495^c^	0.564^c^	0.308^c^
cFIBβ+ (n = 190)	64	112 ± 93	0.4 ± 0.5	150 ± 79	113 ± 124
cFIBβ− (n = 97)	56	94 ± 81	0.3 ± 0.5	137 ± 79	97 ± 72
*P*	0.195	0.108^c^	0.191^c^	0.109^c^	0.229^c^
cVIM+ (n = 92)	65	114 ± 101	0.3 ± 0.4	154 ± 85	120 ± 164
cVIM− (n = 195)	60	102 ± 84	0.4 ± 0.5	142 ± 76	102 ± 70
*P*	0.37	0.508^c^	0.604^c^	0.247^c^	0.979^c^
CEP‐1+ (n = 95)	67	122 ± 98	0.4 ± 0.5	156 ± 81	122 ± 158
CEP‐1− (n = 192)	59	98 ± 84	0.3 ± 0.5	141 ± 78	101 ± 73
*P*	0.21	0.022^c^	0.221^c^	0.105^c^	0.297^c^
cTNC5+ (n = 145)	67	118 ± 96	0.4 ± 0.4	160 ± 78	118 ± 135
cTNC5− (n = 142)	56	93 ± 81	0.3 ± 0.5	131 ± 78	97 ± 73
*P*	0.068	0.015	0.212^c^	0.001	0.109^c^
cCK13‐1+ (n = 69)	68	120 ± 99	0.4 ± 0.4	161 ± 81	137 ± 183
cCK13‐1− (n = 218)	60	101 ± 86	0.3 ± 0.5	141 ± 78	98 ± 69
*P*	0.231	0.197^c^	0.230^c^	0.050^c^	0.078^c^

a Except where indicated otherwise, chi‐square tests were used to compare frequency distributions of categorical variables. *Pg* = *Porphyromonas gingivalis*; PCR = polymerase chain reaction; anti‐RgpB = anti–R gingipain type B; anti‐*Pi* = anti–*Prevotella intermedia*; anti‐*Fn* = anti–*Fusobacterium nucleatum*; CCP‐2 = cyclic citrullinated peptide 2; cFIBβ = citrullinated fibrinogen β; cVIM = citrullinated vimentin; CEP‐1 = citrullinated α‐enolase peptide 1; cTNC5 = citrullinated tenascin‐C; cCK13‐1 = citrullinated cytokeratin 13–derived peptide 1.

b By Fisher's exact test.

c By *t*‐tests and Wilcoxon's rank sum test.

Anti‐RgpB antibodies have been reported to be significantly elevated in PD 31, 35. We confirmed these observations in our study, where anti‐RgpB levels in PD patients were increased compared to those in patients without PD (mean ± SD 0.53 ± 0.54 μg/ml versus 0.34 ± 0.44 μg/ml; *P* < 0.0001) (Supplementary Figure 3A, available on the *Arthritis & Rheumatology* web site at http://onlinelibrary.wiley.com/doi/10.1002/art.40227/abstract). Moreover, anti‐RgpB levels correlated with anti−*P gingivalis* outer membrane antibody levels (r = 0.458, *P* < 0.001), and were associated with PD disease status (*P* = 0.001) as well as detection of *P gingivalis* DNA by PCR (*P* = 0.005) (Supplementary Table 3). However, anti‐RgpB levels were slightly lower in RA patients compared to OA patients (mean ± SD 0.44 ± 0.50 μg/ml versus 0.36 ± 0.45 μg/ml; *P* = 0.0353), and there was no difference in anti‐RgpB levels between CCP‐2–positive patients and CCP‐2–negative patients (mean ± SD 0.34 ± 0.46 μg/ml versus 0.35 ± 0.44 μg/ml; *P* = 0.4988) (Supplementary Figures 3B and C). In addition, RgpB levels did not differ between ACPA‐negative and ACPA‐positive subsets (Table 4).

## Discussion

In this study, by analysis of the citrullinome of GCF and of periodontal tissue, we identified an overlap between periodontal citrullinated peptides and citrullinated peptides that are recognized by autoantibodies in RA. This included a previously unreported modified peptide of CK‐13 (cCK13‐1), which we detected in gingival fluid, and which exhibited elevated antibody responses in patients with RA. Comparing anti–cCK13‐1 antibody levels with levels of other well‐established ACPAs in RA revealed that 2 different sets of antibody fine specificities exist that are differentially associated with RA risk factors.

Previous studies demonstrated the presence of citrullinated proteins in inflamed periodontal tissue by immunohistochemical staining 14, 15. However, that approach cannot reveal the identity of these proteins. Using mass spectrometry, we identified several citrullinated peptides in GCF and periodontal tissue. Notably, a number of citrullinated residues that we detected in periodontal samples have previously been identified in synovial fluid from RA patients (R35 in fibrinogen α [32], R715 in α_2_‐macroglobulin, R450 and R304 in vimentin, and R93 in histone H2B type 2‐E [33]). In addition, we detected peptides with citrullinated residues at position R4 in histone 2A as well as at positions R69 and R71 in vimentin, that are described as autoantibody targets in RA 29, 34. Citrulline 71 in vimentin was also recently detected in lung tissue samples, including those from smokers and patients with chronic obstructive pulmonary disease 27.

Our GCF citrullinome shares some commonalities with the findings in the study by Konig et al 17, who also identified citrullinated peptides of histone 2A and cytokeratin type II in GCF from PD patients. There are also many differences between the studies, which is perhaps not surprising given the nature of mass spectrometry analysis and the small number of samples examined. However, the findings of both studies are consistent with an emerging consensus that citrullination occurs in samples from healthy subjects, with an increase in the range of substrates and extent of protein citrullination in tissues that are more severely inflamed 27, 36, 37. This, in turn, supports the hypothesis that up‐regulation of citrullination by inflammation takes part in driving the ACPA response.

Our analysis also identified unique citrullinated proteins not described so far. Two of these peptides (cCK13‐1 and cCK13‐2), derived from CK‐13, were analyzed in more detail. CK‐13 is an intermediate filament protein that is expressed in the gastrointestinal tract, saliva, and oral mucosa, but not in skin (The Human Protein Atlas [http://www.proteinatlas.org] and Proteomics DB [http://www.proteomicsdb.org]), suggesting that detection of these peptides was not due to contamination of mass spectrometry samples. No antibodies recognizing cCK13‐2 were detected; however, 24% of RA patients had ACPAs against cCK13‐1 (antibody specificity 98%). Both peptides share the same sequence, differing only in the position of one citrullinated arginine residue, underlining the importance of the neighboring amino acids for the specificity of the antibody response. The reactivity toward cCK13‐1 was lower than that toward other ACPA antigens, such as cFIBβ, CEP‐1, cVIM, and cTNC5, but was citrulline‐specific as no enhanced antibody reactivity against the arginine control peptide was detected in RA sera, and 99% of patients who were positive for cCK13‐1 were also positive for CCP‐2.

Data in Figure 2 indicate that combined analysis of anti−cCK13‐1 antibodies with other antibody subtypes, for example anti–CEP‐1, would increase sensitivity, although any benefit gained would likely be offset by a reduction in specificity. Although no cross‐reactivity between anti−cCK13‐1 antibodies and cTNC5 was observed, cCK13‐1 antibody levels correlated well with cTNC5 antibody levels, and almost all RA patients who were positive for cCK13‐1 were also positive for cTNC5. In addition, both cTNC5 and cCK13‐1 peptide sequences lie close to the C‐terminal end of their corresponding protein, making it intriguing to speculate that both peptides may be generated by a common mechanism or target enzyme.

Citrullinated CK‐13 was only detected in GCF, and not in PD tissue. Differences in the citrullinome of GCF and PD tissue may arise from underlying biologic variation between the 2 sites. For example, CK‐13 is predominantly expressed in noncornified stratified epithelia such as mucosal membranes, but levels are most abundant in saliva, where it is found at levels 3–6 orders of magnitude higher than in tissues (Proteomics DB). Soluble CK‐13 in GCF may also exhibit greater accessibility to PADs than that constrained or masked within cells or within tissue. Proteomics analysis of tissue and fluid also each face distinct technical challenges, for example, sample dilution, varying dynamic ranges of protein abundance, and ease of protein isolation, which may account for differences in peptide profiles detected at each site. Finally, analysis of a single tissue sample precludes generalization that cCK13 is not formed in inflamed periodontal tissue.

Despite finding cCK13‐1 in patients with PD alone, or with coexisting RA and PD, and not in RA patients without PD, larger sample sizes are required to determine if this antigen arises specifically in GCF during PD. The lack of association of anti−cCK13‐1 antibodies with PD suggests that while GCF may provide a rich source of cCK13‐1, it is not the only source of this antigen. Indeed, CK‐13 is also expressed in the lung and gut (Proteomics DB), which raises the intriguing possibility that citrullination of this peptide in the mucosal epithelia of these organs may also link dysbiosis at these sites with autoantibody generation in RA.

Taken together, these data demonstrate that citrullination occurs in periodontal tissue in patients with PD and that these citrullinated peptides can serve as autoantigens in RA, suggesting that citrullinated proteins generated in extraarticular tissues such as the inflamed periodontium may play a role in breaching tolerance in RA.

Because of the small number of GCF samples, it was not possible to analyze whether the frequency of citrullination is higher in patients with PD and/or RA compared to healthy controls. However, little citrullination has been reported in GCF from healthy subjects, compared to proteins in GCF from PD patients, which are heavily citrullinated 17. In our study, we detected citrullinated peptides in GCF from healthy controls. Citrullination is often associated with inflammation 37, but low levels of protein citrullination have also been reported in tissue from healthy lungs, lymph nodes, skeletal muscle, and kidney 27. We predominantly detected internally citrullinated peptides, suggesting that these peptides were citrullinated by the action of endogenous human PADs 36, 38, and not by PPAD, which preferentially citrullinates C‐terminal arginines 18, 19, 39. These findings are consistent with those of a previous study that identified only peptides with endocitrullination in GCF samples from PD patients 17.

Environmental and genetic risk factors are involved in driving the autoimmune response toward citrullinated peptides 40, 41, 42. As described previously 43, we found that CEP‐1 and cVIM were associated with the HLA−DRB1 shared epitope, and cFIBβ and CEP‐1 were associated with smoking. We previously reported that cTNC5 antibodies were significantly associated with HLA−DRB1 shared epitope status 7, and here we describe a close to significant association (*P* = 0.085). Although we observed no correlation of cVIM with smoking, in contrast to other studies 43, the lack of association of cTNC5 with smoking is consistent with data from other cohorts showing weak 7 or no 44 links to smoking for this ACPA subset. Neither of these RA risk factors were associated with cCK13‐1 antibodies. These data demonstrate that ACPA fine specificities are differentially associated with environmental and genetic RA risk factors, which in some cases are stronger than associations observed with CCP‐2.

The cFIBβ antibodies were associated with PD in general, while anti–CEP‐1 and anti–cTNC5 were correlated with anti−*P gingivalis* outer membrane antibodies. However, we observed no association of these antibodies with PCR‐based detection of subgingival *P gingivalis*. Although CEP‐1 has previously been discussed as a candidate autoantigen involved in the etiology of RA, since antibodies to citrullinated *P gingivalis* α‐enolase cross‐react with antibodies to CEP‐1 and therefore may be involved in breaching tolerance in RA 45, this is the first study to link ACPA reactivities with other periodontal pathogens. Intriguingly, both cTNC5 and cCK13‐1 were associated with antibodies reactive with *P intermedia*. Although the correlations were weak numerically, the statistical associations were highly significant. Relatively small correlations with significant *P* values are common for the sample size of the cohort examined here, and while a small correlation does not mean that the 2 variables are not related, it does mean that most (but not all) of the value of one variable is not determined by the value of the other variable. These data therefore provide supporting evidence of linked pathologic processes, but also suggest that additional contributing factors underlie disease pathology.

Infection with *P intermedia*, unlike *P gingivalis,* does not exacerbate collagen‐induced arthritis 46, and, given that *P intermedia* does not express a PAD, this suggests that infection with this bacterium may drive the generation of distinct ACPA fine specificities in a manner quite different from *P gingivalis*. For example, it may facilitate human PAD activity in the periodontium through induction of local inflammation. Activated neutrophils are suggested as a source of citrullinated antigens 47 and a recent publication demonstrated how nucleases from *P intermedia* can degrade neutrophil extracellular traps, which could lead to increased periodontal pathogenicity 48 and release of endogenous PADs 49. A recent study also demonstrated that *Aggregatibacter actinomycetemcomitans*, but not *P gingivalis*,* F nucleatum*, or *P intermedia*, could induce citrullination of intracellular proteins in neutrophils, and that antibodies against *A actinomycetemcomitans* are associated with citrullinated intracellular proteins but not citrullinated extracellular proteins 17. Taken together, these data demonstrate that infection with different periodontal pathogens may drive the generation of different ACPA fine specificities.

Anti‐RgpB antibody levels, used as an additional marker for *P gingivalis* infection as in previous publications 35, 50, 51, correlated well with anti−*P gingivalis* antibody levels and were significantly higher in RA patients with PD than in RA patients without PD. However, we observed no association between any ACPA and these antibodies. In contrast to previous studies in 2 well‐documented Swedish cohorts, we observed no association of anti‐RgpB with CCP‐2–positive RA, nor did we observe elevated anti‐RgpB levels in RA patients compared to OA patients, or in CCP‐2–positive RA patients compared to CCP‐2–negative RA patients 35, 51. Our data are consistent with previous studies showing no association of anti‐RgpB antibodies with RA or pre‐RA 31, 50 and no difference in anti–*P gingivalis* outer membrane antibody levels between RA and non‐RA cohorts 12. Our findings were also consistent with a previous study that showed that anti–*P gingivalis* antibody levels were lower, though not significantly, in RA patients than in subjects without RA 52. These different results may be due to differences in the cohorts analyzed, for example, differences in disease duration and severity, methods of antibody measurement used, the age and sex of the population studied, or, perhaps most importantly, the use of healthy control groups versus the OA comparator group used here.

In summary, our study has identified 2 groups of ACPA fine specificities, each associated with different environmental and genetic RA risk factors. Antibodies to a novel peptide, cCK13‐1, correlated strongly with anti‐cTNC5, both of which were linked to a serologic response to infection with the periodontal pathogen *P intermedia*. The second group, comprising anti−CEP‐1, anti‐cFIBβ, and anti‐cVIM, was predominantly associated with smoking and the shared epitope, consistent with the findings of previous studies. The identification of further distinct ACPA fine specificities, and the analysis of ACPA subsets in the context of the complex changes that occur in the oral microbiota during PD, will likely reveal further etiologic subsets of RA. This information will be important in helping understand different mechanisms by which tolerance can be breached to drive autoimmunity in this heterogeneous disease.

## Author Contributions

All authors were involved in drafting the article or revising it critically for important intellectual content, and all authors approved the final version to be published. Dr. Midwood had full access to all of the data in the study and takes responsibility for the integrity of the data and the accuracy of the data analysis.

### Study conception and design

Schwenzer, Quirke, Fischer, Venables, Payne, Mikuls, Midwood.

### Acquisition of data

Schwenzer, Quirke, Marzeda, Wong, Montgomery, Eick, Gawron, Chomyszyn‐Gajewska, Łazarz‐Bartyzel, Davis, Fischer, Venables, Payne, Mikuls, Midwood.

### Analysis and interpretation of data

Schwenzer, Quirke, Wong, Sayles, Potempa, Kessler, Fischer, Venables, Payne, Mikuls, Midwood.

## Supporting information

Supinfo
