# Supplementary material for: Association of Distinct Fine Specificities of Anti−Citrullinated Peptide Antibodies With Elevated Immune Responses to Prevotella intermedia in a Subgroup of Patients With Rheumatoid Arthritis and Periodontitis
Source: Arthritis Rheumatol. 2017 Oct 30;69(12):2303–13. doi: 10.1002/art.40227 (PMC5711558; doi:10.1002/art.40227)
Supplement: Supplementary file 1 — Supinfo [file ART-69-2303-s001.docx]

**Supplementary Tables**

| \| **Table 1.** Citrullinated peptides identified in GCF from healthy controls (n=2), patients with both PD and RA (PD&RA) (n=3) or patients with PD (n=2) or RA (n=1) alone.   \| **Accession Number, Protein and identified citrullinated peptides** \| **Control**  n=2 \| **PD&RA**  n=3 \| **PD**  n=2 \| **RA**  n=1 \| \| --- \| --- \| --- \| --- \| --- \| \| **Q9UBG3, Cornulin** \|  \|  \|  \|  \| \| _119_SGTEVG**(cit)**AGKGQHYEGSSHR_138_ ^(ii)^ \| 0 \| 1 \| 0 \| 0 \| \| _437_VVGEEWVDDHSR**(cit)**ETV_451_ ^(i)^ \| 1 \| 0 \| 0 \| 0 \| \| _452_IL**(cit)**LDQGNLHTSV_464_ ^(i)^ \| 1 \| 0 \| 0 \| 0 \| \| _465_SSAQGQDAAQSEEK**(cit)**GI_481_ ^(i)^ \| 1 \| 0 \| 0 \| 0 \| \| **P02671, Fibrinogen α** \|  \|  \|  \|  \| \| _20_ADSGEGDFLAEGGGV**(cit)_35_** ^(i)^ \| 0 \| 1 \| 0 \| 0 \| \| **Q96KK5, Histone 2A** \|  \|  \|  \|  \| \| _1_MSG**(cit)**GKQGGKARAKAKTR_18_ ^(i)^ \| 1 \| 2 \| 1 \| 0 \| \| P13646, Keratin, type I cytoskeletal 13 \|  \|  \|  \|  \| \| _5_LQSSSASYGGGFGGGSCQLGGG**(cit)**GVSTCSTR_35_ ^(i)^ \| 1 \| 0 \| 0 \| 0 \| \| _326_RTLQGLEIELQSQLSMKAGLENTVAETEC**(cit)**_355_ ^(ii)^ \| 1 \| 0 \| 0 \| 0 \| \| _407_SLLEGQDAKMIGFPSSAGSVSP**(cit)**R_430_ ^(i)^ \| 1 \| 0 \| 0 \| 0 \| \| _430_STSVTTTSSASVTTTSNASG**(cit)**R_451_ ^(i, ii)^ \| 1 \| 1 \| 2 \| 0 \| \| _451_**(cit)**TSDV**(cit)**RP_458_ ^(i, ii)^ \| 1 \| 2 \| 1 \| 0 \| \| _451_**(cit)**TSDVR**(cit)**P_458_ ^(i, ii)^ \| 1 \| 2 \| 1 \| 0 \| \| _452_TSDV**(cit)**RP_458_ ^(ii)^ \| 1 \| 1 \| 1 \| 1 \| \| _452_TSDVR**(cit)**P_458_ ^(ii)^ \| 1 \| 1 \| 0 \| 0 \| \| **Q04695, Keratin, type I cytoskeletal 17** \|  \|  \|  \|  \| \| _425_EQVHQTT**(cit)_432_** ^(ii)^ \| 0 \| 1 \| 0 \| 0 \| \| P04264, Keratin, type II cytoskeletal 1 \|  \|  \|  \|  \| \| _31_TTSSST**(cit)(cit)**SGGGGGR_44_ ^(ii)^ \| 1 \| 2 \| 0 \| 1 \| \| **P19013, Keratin, type II cytoskeletal 4** \|  \|  \|  \|  \| \| _5_QQCV**(cit)**GGPR_13_ ^(ii)^ \| 0 \| 1 \| 0 \| 0 \| \| **P02538/P04259/P48668, Keratin, type II cytoskeletal 6A/B/C** \|  \|  \|  \|  \| \| _2_ASTSTTI**(cit)**SHSSSR_15_ ^(ii)^ \| 1 \| 1 \| 0 \| 1 \| \| _10_SHSSS**(cit)(cit)**GFSANSAR_24_ ^(i, ii)^ \| 1 \| 2 \| 2 \| 0 \| \| _22_SA**(cit)**LPGVS**(cit)**SGF_33_ ^(i)^ \| 1 \| 0 \| 0 \| 0 \| \| _41_S**(cit)**GSGGLGGACGGAGFGSR_59_ ^(ii)^ \| 2 \| 1 \| 1 \| 0 \| \| _348_S**(cit)**AEAESWYQTK_359_ ^(ii)^ \| 0 \| 1 \| 0 \| 0 \| \| _468_LLEGEEC**(cit)_475_** ^(ii)^ \| 1 \| 1 \| 0 \| 0 \| \| _550_YTTTSSSS**(cit)**K_560_ ^(i, ii)^ \| 2 \| 1 \| 1 \| 1 \|   Key: (i) GCF with no tryptic digest, (ii) tryptic digest of GCF solution \| \| --- \| --- \| --- \| --- \| --- \| --- \| --- \| --- \| --- \| --- \| --- \| --- \| --- \| --- \| --- \| --- \| --- \| --- \| --- \| --- \| --- \| --- \| --- \| --- \| --- \| --- \| --- \| --- \| --- \| --- \| --- \| --- \| --- \| --- \| --- \| --- \| --- \| --- \| --- \| --- \| --- \| --- \| --- \| --- \| --- \| --- \| --- \| --- \| --- \| --- \| --- \| --- \| --- \| --- \| --- \| --- \| --- \| --- \| --- \| --- \| --- \| --- \| --- \| --- \| --- \| --- \| --- \| --- \| --- \| --- \| --- \| --- \| --- \| --- \| --- \| --- \| --- \| --- \| --- \| --- \| --- \| --- \| --- \| --- \| --- \| --- \| --- \| --- \| --- \| --- \| --- \| --- \| --- \| --- \| --- \| --- \| --- \| --- \| --- \| --- \| --- \| --- \| --- \| --- \| --- \| --- \| --- \| --- \| --- \| --- \| --- \| --- \| --- \| --- \| --- \| --- \| --- \| --- \| --- \| --- \| --- \| --- \| --- \| --- \| --- \| --- \| --- \| --- \| --- \| --- \| --- \| --- \| --- \| --- \| --- \| --- \| --- \| --- \| --- \| --- \| --- \| --- \| --- \| --- \| --- \| --- \| --- \| --- \| --- \| --- \| --- \| --- \| --- \| --- \| --- \| --- \| --- \| --- \| --- \| --- \| --- \| --- \| --- \| --- \| --- \| --- \| \| GCF, gingival crevicular fluid; Cit, citrulline; PD, periodontitis; RA, rheumatoid arthritis. \| \|  \| |
| --- | --- | --- | --- | --- | --- | --- | --- | --- | --- | --- | --- | --- | --- | --- | --- | --- | --- | --- | --- | --- | --- | --- | --- | --- | --- | --- | --- | --- | --- | --- | --- | --- | --- | --- | --- | --- | --- | --- | --- | --- | --- | --- | --- | --- | --- | --- | --- | --- | --- | --- | --- | --- | --- | --- | --- | --- | --- | --- | --- | --- | --- | --- | --- | --- | --- | --- | --- | --- | --- | --- | --- | --- | --- | --- | --- | --- | --- | --- | --- | --- | --- | --- | --- | --- | --- | --- | --- | --- | --- | --- | --- | --- | --- | --- | --- | --- | --- | --- | --- | --- | --- | --- | --- | --- | --- | --- | --- | --- | --- | --- | --- | --- | --- | --- | --- | --- | --- | --- | --- | --- | --- | --- | --- | --- | --- | --- | --- | --- | --- | --- | --- | --- | --- | --- | --- | --- | --- | --- | --- | --- | --- | --- | --- | --- | --- | --- | --- | --- | --- | --- | --- | --- | --- | --- | --- | --- | --- | --- | --- | --- | --- | --- | --- | --- | --- | --- | --- | --- |

**Table 2.** Citrullinome of PD Tissue. PD, periodontitis; Cit, citrulline.

| **Protein Accession** | **Protein name** | **Peptide** | **Start** | **End** |
| --- | --- | --- | --- | --- |
| O43707 | Alpha-actinin-4 | R.LSN-cit-PAFMPSEGK.M | 366 | 378 |
| O60814 | Histone H2B type 1-K | R.STITS-cit-EIQTAVR.L | 88 | 100 |
| P01023 | Alpha-2-macroglobulin | F.YESDVMG-cit-GHAR.L | 708 | 719 |
| P02042 | Hemoglobin subunit delta | R.LLVVYPWTQ-cit-FFESFGDLSSPDAVMGNPK.V | 32 | 60 |
| P02545-2 | Prelamin-A/C | K.-cit-KLESTESR.S | 419 | 427 |
|  |  | R.KLESTES-cit-SSFSQHAR.T | 420 | 435 |
|  |  | R.S-cit-GRASSHSSQTQGGGSVTK.K | 398 | 417 |
|  |  | R.RAT-cit-SGAQASSTPLSPTR.I | 8 | 25 |
| P02647 | Apolipoprotein A-I | Q.DEPPQSPWD-cit-VKDLATVYVDVLKDSGR.D | 25 | 51 |
| P02671 | Fibrinogen alpha chain | R.MELE-cit-PGGNEITRGGSTSYGTGSETESPRNPS.S | 259 | 290 |
|  |  | R.MELERPGGNEIT-cit-GGSTSYGTGSETESPRNPS.S | 259 | 290 |
| P02751 | Fibronectin | L.PGTEYVVSVSSVYEQHESTPL-cit-G-cit-QKTGLDSPTGIDFSDITANSFTVHWIAPR.A | 1330 | 1382 |
| P02768 | Serum albumin | K.NYAEAKDVFLGMFLYEYA-cit-.R | 342 | 360 |
|  |  | K.YLYEIAR-cit-HPYFYAPELLFFAKR.Y | 162 | 184 |
|  |  | R.HPYFYAPELLFFAK-cit-YK.A | 170 | 186 |
|  |  | K.FGE-cit-AFKAWAVAR.L | 230 | 242 |
|  |  | R.LV-cit-PEVDVMCTAFHDNEETFLKKYLYEIAR.R | 139 | 168 |
|  |  | K.YLYEIAR-cit-HPYFYAPELLFFAK.R | 162 | 183 |
|  |  | R.-cit-HPDYSVVLLLR.L | 361 | 372 |
|  |  | R.-cit-HPYFYAPELLFFAKR.Y | 169 | 184 |
|  |  | R.LV-cit-PEVDVMCTAFHDNEETFLKKYLYEIARR.H | 139 | 169 |
| P06396 | Gelsolin | T.AS-cit-GASQAGAPQGR.V | 30 | 43 |
|  |  | A.ATAS-cit-GASQAGAPQGR.V | 28 | 43 |
| P06576 | ATP synthase subunit beta, mitochondrial | K.KGSITSVQAIYVPADDLTDPAPATTFAHLDATTVLS-cit-.A | 351 | 387 |
| P06899 | Histone H2B type 1-J | R.STITS-cit-EIQTAVR.L | 88 | 100 |
| P08670 | Vimentin | K.TVET-cit-DGQVINETSQHHDDLE | 446 | 466 |
|  |  | R.SSAV-cit-L-cit-SSVPGVR.L | 65 | 78 |
|  |  | K.FADLSEAAN-cit-NNDALR.Q | 295 | 310 |
| P09651 | Heterogeneous nuclear ribonucleoprotein A1 | R.S-cit-GFGFVTYATVEEVDAAMNARPHKVDGR.V | 54 | 82 |
| P11021 | 78 kDa glucose-regulated protein | K.VYEGE-cit-PLTKDNHLLGTFDLTGIPPAPR.G | 465 | 492 |
| P19971 | Thymidine phosphorylase | R.VAAALDDGSALG-cit-FERMLAAQGVDPGLAR.A | 330 | 358 |
| P21333 | Filamin-A | R.FLP-cit-EEGPYEVEVTYDGVPVPGSPFPLEAVAPTKPSKVK.A | 1033 | 1071 |
|  |  | R.AGGPGLERAEAGVPAEFSIWT-cit-.E | 2243 | 2264 |
|  |  | K.GLVEPVDVVDNADGTQTVNYVPS-cit-EGPYSISVLYGDEEVPRSPFK.V | 1492 | 1536 |
| P22626 | Heterogeneous nuclear ribonucleoproteins A2/B1 | R.S-cit-GFGFVTFSSMAEVDAAMAARPHSIDGRVVEPK.R | 61 | 94 |
| P22626 | Heterogeneous nuclear ribonucleoproteins A2/B1 | R.S-cit-GFGFVTFSSMAEVDAAMAARPHSIDGRVVEPKR.A | 61 | 95 |
| P26641 | Elongation factor 1-gamma | R.KLDPGSEETQTLV-cit-EYFSWEGAFQHVGKAFNQGK.I | 401 | 434 |
| P33778 | Histone H2B type 1-B | R.STITSR-cit-EIQTAVR.L | 88 | 100 |
| P35609 | Alpha-actinin-2 | R.ISN-cit-PAFMPSEGK.M | 354 | 366 |
| P35609-2 | Alpha-actinin-2 | R.ISN-cit-PAFMPSEGK.M | 354 | 366 |
| P46779 | 60S ribosomal protein L28 | R.NSF-cit-YNGLIHRK.T | 36 | 47 |
| P52790 | Hexokinase-3 | R.AQLQQIQASLLGSMEQAL-cit-GQASPAPAVR.M | 45 | 73 |
| P53999 | Activated RNA polymerase II transcriptional coactivator p15 | K.TGETS-cit-ALSSSK.Q | 42 | 53 |
| P57053 | Histone H2B type F-S | R.STITS-cit-EIQTAVR.L | 88 | 100 |
| P58876 | Histone H2B type 1-D | R.STITS-cit-EIQTAVR.L | 88 | 100 |
| P62249 | 40S ribosomal protein S16 | K.-cit-GNGLIKVNGRPLEMIEPR.T | 27 | 45 |
| P62807 | Histone H2B type 1-C/E/F/G/I | R.STITS-cit-EIQTAVR.L | 88 | 100 |
| P68871 | Hemoglobin subunit beta | R.LLVVYPWTQ-cit-FFESFGDLSTPDAVMGNPK.V | 32 | 60 |
|  |  | K.SAVTALWGKVNVDEVGGEALGRLLVVYPWTQ-cit-FFESFGDLSTPDAVMGNPK.V | 10 | 60 |
|  |  | K.SAVTALWGKVNVDEVGGEALG-cit-LLVVYPWTQR.F | 10 | 41 |
|  |  | R.LLVVYPWTQ-cit-FFESFGDLSTPDAVMGNPKVK.A | 32 | 62 |
|  |  | R.LLVVYPWTQ-cit-FFESFGDLSTPDAVMGNPKVKAHGK.K | 32 | 66 |
|  |  | R.LLVVYPWTQ-cit-FFESFGDLSTPDAVMGNPK.V | 32 | 60 |
|  |  | R.LLVVYPWTQ-cit-FFESFGDLSTPDAVMGNPKVKA.H | 32 | 63 |
|  |  | K.VNVDEVGGEALGRLLVVYPWTQ-cit-FFESFGDLSTPDAVMGNPK.V | 19 | 60 |
| P69905\| HBA_HUMAN | Hemoglobin subunit alpha | K.AAWGKVGAHAGEYGAEALE-cit-MFLSFPTTK.T | 13 | 41 |
|  |  | K.AAWGKVGAHAGEYGAEALE-cit-MFLSFPTTKTYFPHFDLSHGSAQVK.G | 13 | 57 |
|  |  | K.VGAHAGEYGAEALE-cit-MFLSFPTTKTYFPHFDLSHGSAQVK.G | 18 | 57 |
|  |  | K.VGAHAGEYGAEALE-cit-MFLSFPTTK.T | 18 | 41 |
|  |  | K.VGAHAGEYGAEALE-cit-.M | 18 | 32 |
| P02042\| HBD_HUMAN | Hemoglobin subunit delta | R.LLVVYPWTQ-cit-FFESFGDLSSPDAVMGNPK.V | 32 | 60 |
| Q15084 | Protein disulfide-isomerase A6 | R.GSTAPVGGGAFPTIVE-cit-EPWDGRDGELPVEDDIDLSDVELDDLGKDEL | 393 | 440 |
| Q16778 | Histone H2B type 2-E | R.STITS-cit-EIQTAVR.L | 88 | 100 |
| Q16822 | Phosphoenolpyruvate carboxykinase [GTP], mitochondrial | R.TMYVLPFSMGPVGSPLS-cit-.I | 156 | 173 |
|  |  | R.TMYVLPFSMGPVGSPLS-cit-.I | 156 | 173 |
| Q58FF3 | Putative endoplasmin-like protein | F.QSSHHPADITSLHQDVE-cit-MK.E | 170 | 189 |
| Q71U36 | Tubulin alpha-1A chain | R.AVFVDLEPTVIDEVRTGTY-cit-QLFHPEQLITGKEDAANNYAR.G | 65 | 105 |
| Q7KZF4 | Staphylococcal nuclease domain-containing protein 1 | R.YGDFRADDADEFGYS-cit- | 895 | 910 |
| Q99715 | Collagen alpha-1(XII) chain | R.NVQVYNPTPNSLDV-cit-WDPAPGPVLQYRVVYSPVDGTRPSESIVVPGNTR.M | 1939 | 1987 |
| Q9BQE3 | Tubulin alpha-1C chain | R.AVFVDLEPTVIDEVRTGTY-cit-QLFHPEQLITGKEDAANNYAR.G | 65 | 105 |
| tr\|A0A075B6N7 | Submitted name: Ig alpha-2 chain C region | K.YLTWAS-cit-QEPSQGTTTYAVTSILR.V | 263 | 286 |
| P01877 | Ig alpha-2 chain C region | R.WLQGSQELP-cit-EKYLTWASRQEPSQGTTTFAVTSILR.V | 251 | 286 |
| tr\|B7WNR0 | Submitted name: Serum albumin | K.NYAEAKDVFLGMFLYEYA-cit-.R | 227 | 245 |
|  |  | L.YLYEIAR-cit-HPYFYAPELLFFAKR.Y | 47 | 69 |
|  |  | R.HPYFYAPELLFFAK-cit-YK.A | 55 | 71 |
|  |  | K.FGE-cit-AFKAWAVAR.L | 115 | 127 |
| tr\|D6RGG3 | Submitted name: Collagen alpha-1(XII) chain | R.NVQVYNPTPNSLDV-cit-WDPAPGPVLQYRVVYSPVDGTRPSESIVVPGNTR.M | 1939 | 1987 |
| tr\|F5H5D3 | Submitted name: Tubulin alpha-1C chain | R.AVFVDLEPTVIDEVRTGTY-cit-QLFHPEQLITGKEDAANNYAR.G | 135 | 175 |
| tr\|F8W6I7 | Submitted name: Heterogeneous nuclear ribonucleoprotein A1 | R.S-cit-GFGFVTYATVEEVDAAMNARPHKVDGR.V | 54 | 82 |
| tr\|H0YM31 | Submitted name: Phosphoenolpyruvate carboxykinase [GTP], mitochondrial | R.TMYVLPFSMGPVGSPLS-cit-.I | 168 | 185 |
| tr\|Q5JVS8 | Submitted name: Vimentin | K.FADLSEAAN-cit-NNDALR.Q | 121 | 136 |
| tr\|Q5TCI8 | Submitted name: Prelamin-A/C | K.-cit-KLESTESR.S | 338 | 346 |
|  |  | R.KLESTES-cit-SSFSQHAR.T | 339 | 354 |
|  |  | R.S-cit-GRASSHSSQTQGGGSVTK.K | 317 | 336 |
| tr\|U3KQK0 | Histone H2B | R.STITS-cit-EIQTAVR.L | 88 | 100 |
| P01024 | Complement C3 | G.SPMYSIITPNIL-cit-LESEETMVLEAHDAQGDVPVTVTVHDFPGKK.L | 23 | 66 |
| P0C0L5 | Complement C4-B | R.VTASDPLDTLGSEGALSPGGVASLL-cit-LPR.G | 980 | 1008 |
| P08514 | Integrin alpha-IIb | R.FGSAIAPLGDLD-cit-DGYNDIAVAAPYGGPSGRGQVLVFLGQSEGLR.S | 387 | 431 |
| P13716 | Delta-aminolevulinic acid dehydratase | R.AVDRDVREGADMLMVKPGMPYLDIV-cit-.E | 237 | 262 |
| P21333 | Filamin-A | R.FLP-cit-EEGPYEVEVTYDGVPVPGSPFPLEAVAPTKPSK.V | 1033 | 1069 |
| P22061 | Protein-L-isoaspartate(D-aspartate) O-methyltransferase | R.VQLVVGDG-cit-MGYAEEAPYDAIHVGAAAPVVPQALIDQLKPGGR.L | 136 | 178 |
| P32119 | Peroxiredoxin-2 | K.TDEGIAY-cit-GLFIIDGKGVLRQITVNDLPVGR.S | 120 | 150 |

**Table 3.** Comparisons of anti-RgpB levels in RA patients by categorical variables. RgpB, arginine gingipain B; RA, rheumatoid arthritis.

|  | N | Mean (SD) | p-value |
| --- | --- | --- | --- |
| Smoking status |  |  |  |
| Never | 108 | 0.30 (0.40) |  |
| Former | 124 | 0.37 (0.46) | 0.393^a^ |
| Current | 55 | 0.44 (0.53) |  |
| HLA-DRB1 Shared Epitope |  |  |  |
| Positive | 200 | 0.34 (0.45) | 0.877^b^ |
| Negative | 64 | 0.33 (0.42) |  |
| Periodontitis |  |  |  |
| Positive | 100 | 0.46 (0.50) | 0.001 ^b^ |
| Negative | 187 | 0.30 (0.41) |  |
| Subgingival *P gingivalis* |  |  |  |
| Positive | 174 | 0.45 (0.52) | 0.005 ^b^ |
| Negative | 109 | 0.21 (0.25) |  |
| Anti-*P gingivalis* |  |  |  |
| Spearman Correlation | 287 | r=0.458 | <0.001 |

a – Kruskal-Wallis non-parametric ANOVA

b – Wilcoxon rank sum test

**Supplementary Figures**

**Figure 1.** Mass spectrometry analysis of citrullinated peptides derived from cytokeratin 13 (P13646). The MS/MS spectra of tryptic peptides are shown, where r refers to the citrullinated position. The matched fragment ions of the y-type (red) and b-type (blue) are shown.


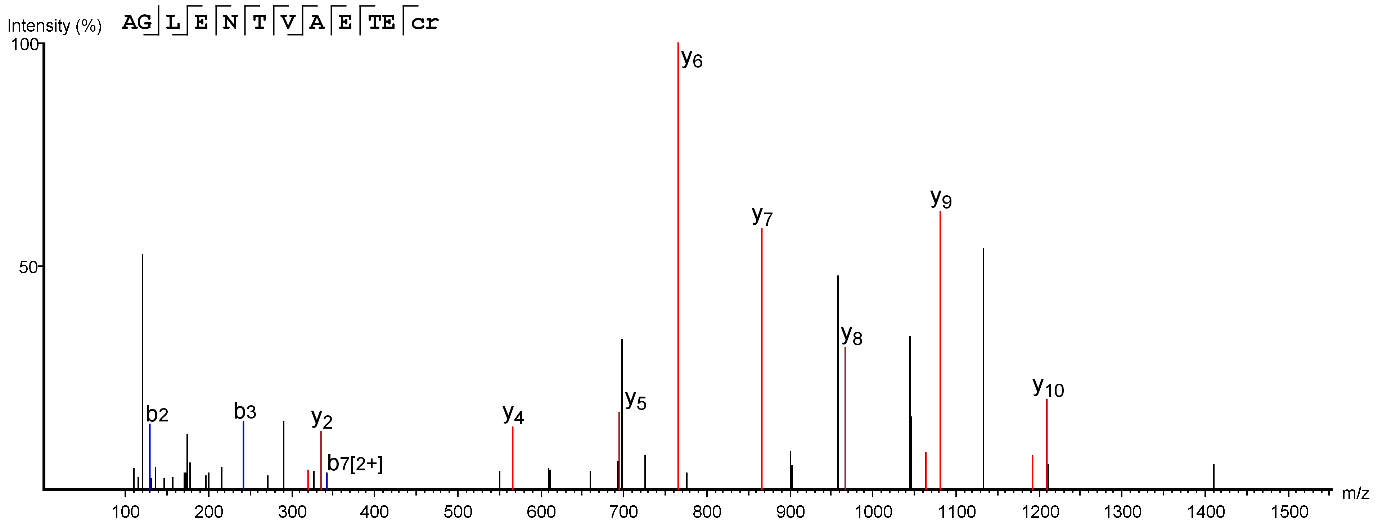

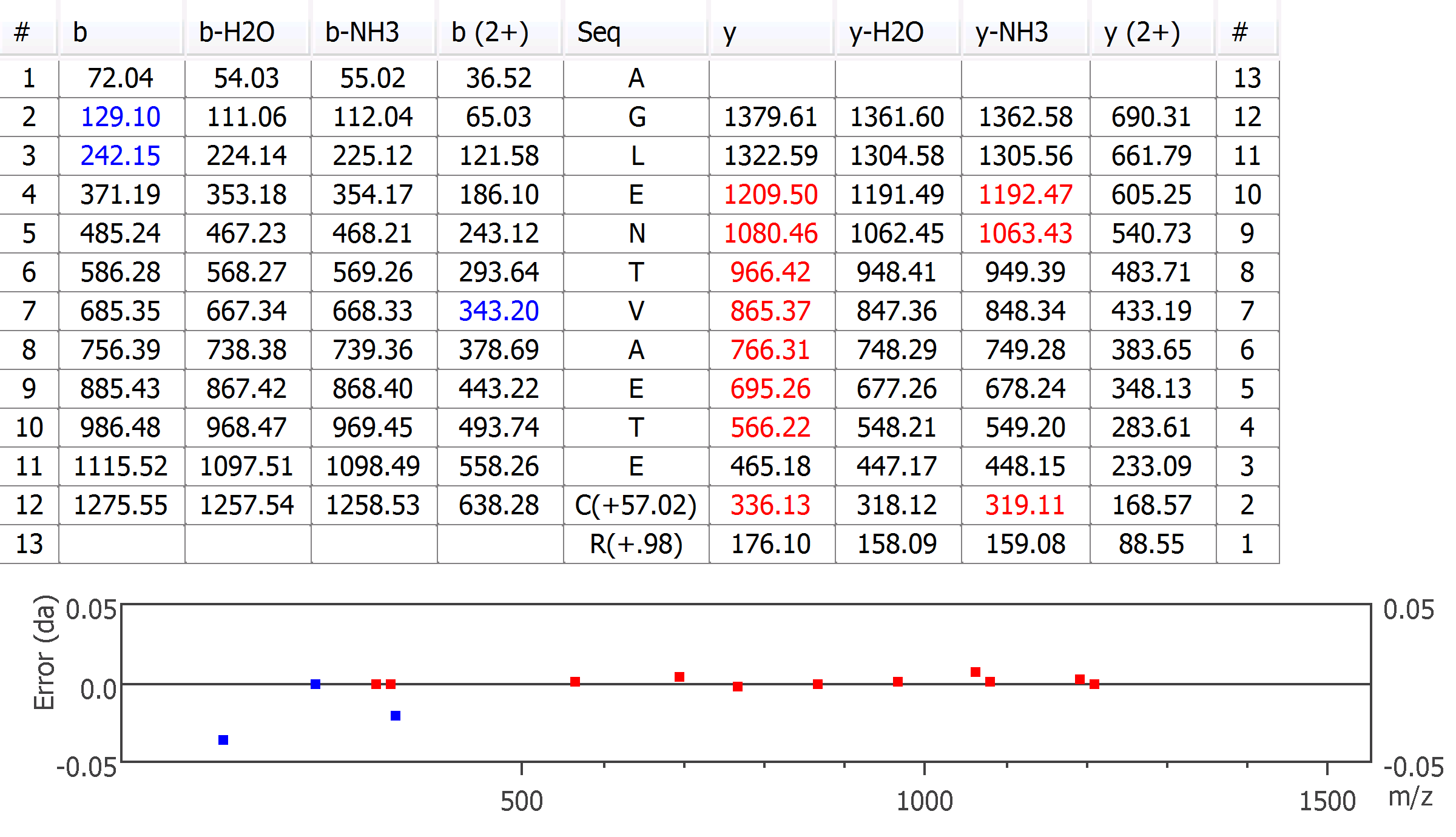


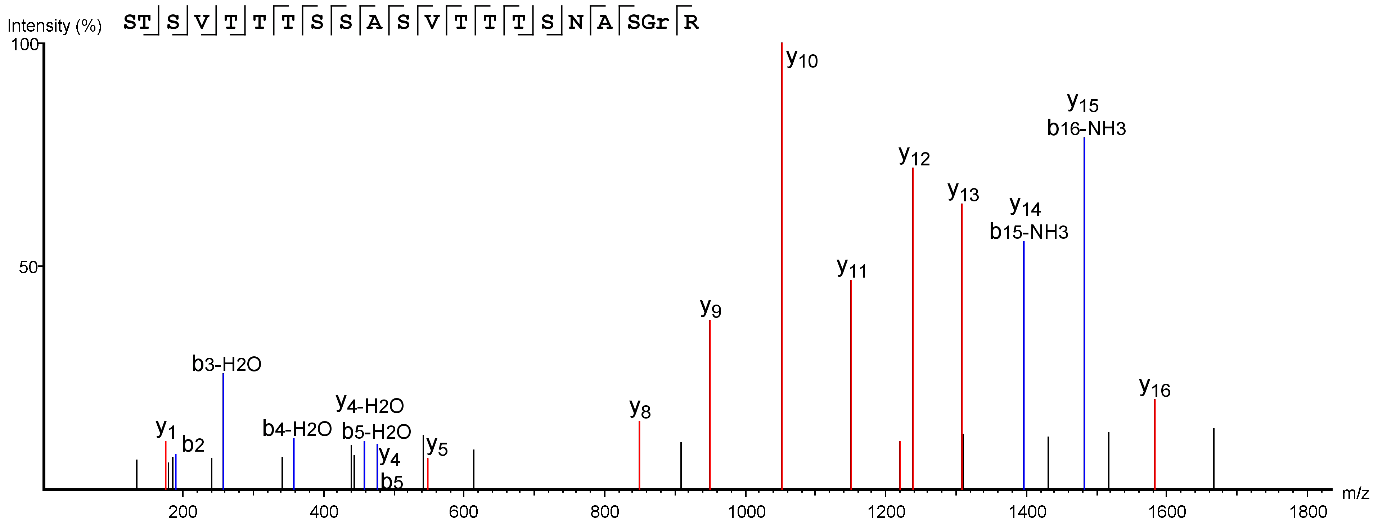

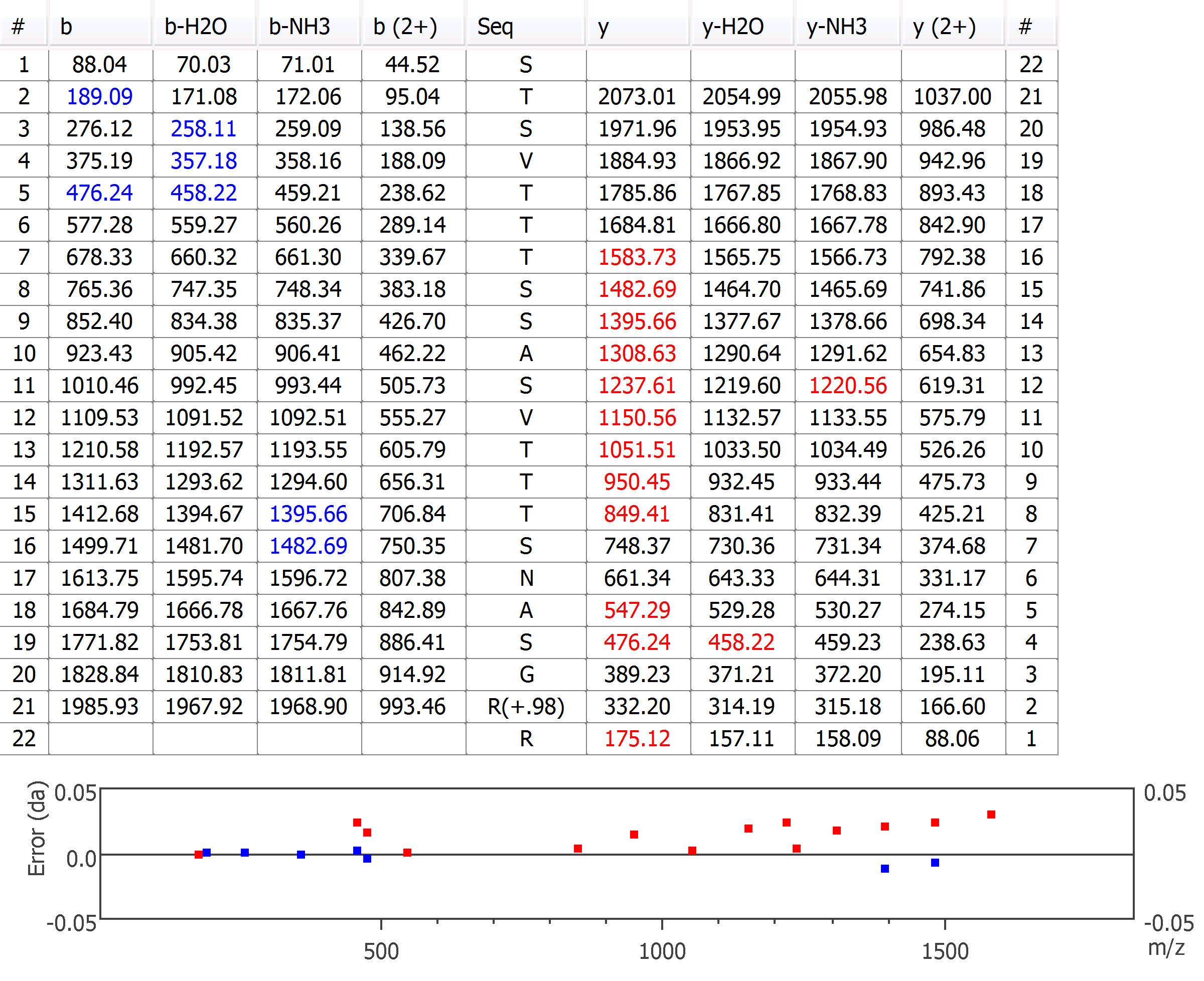


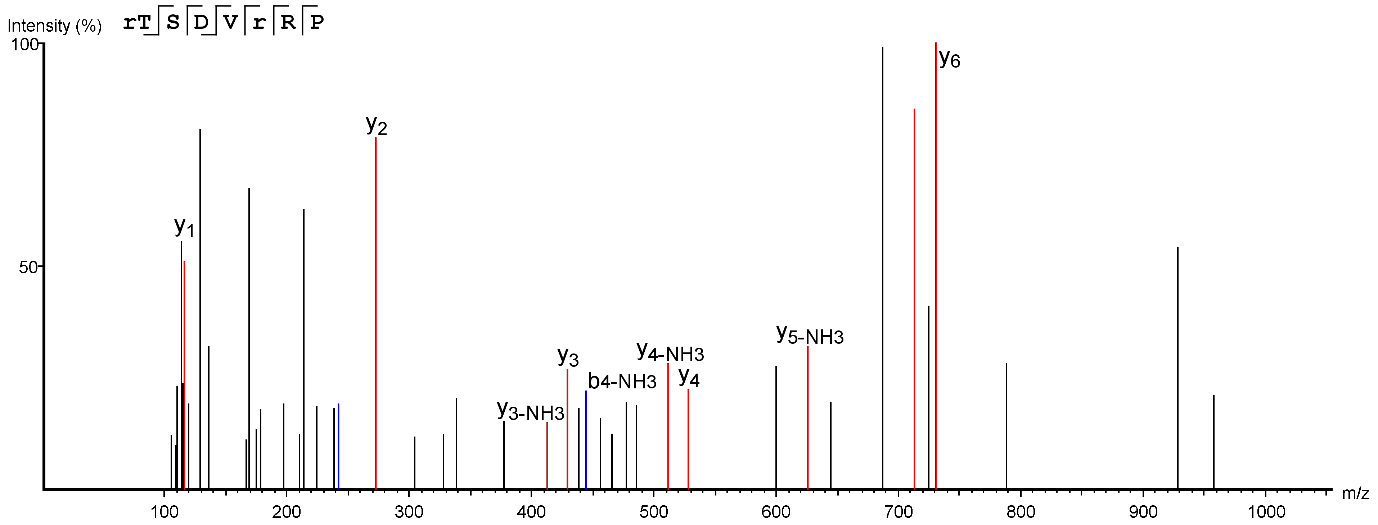


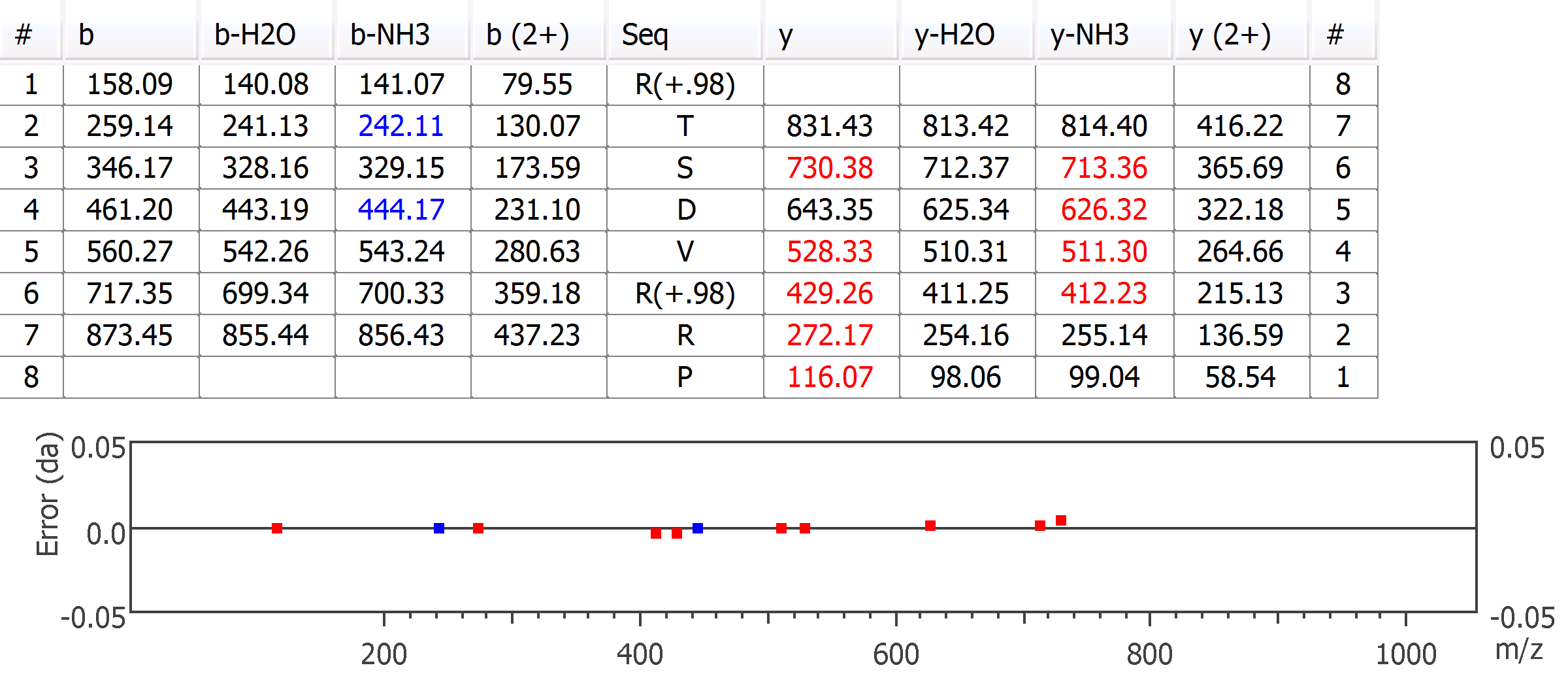


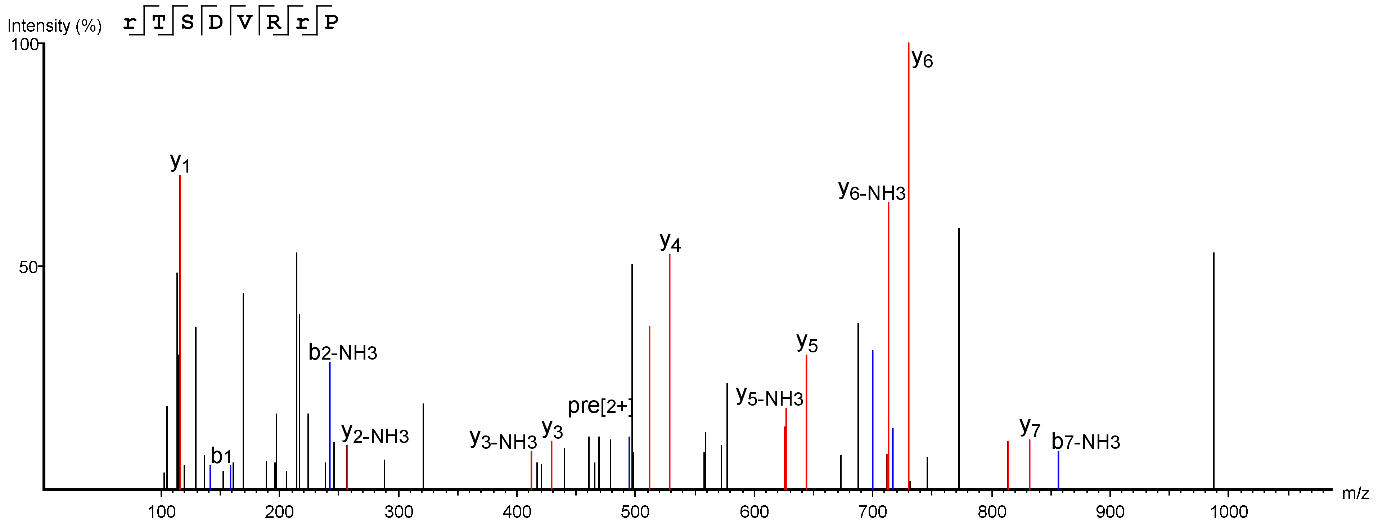

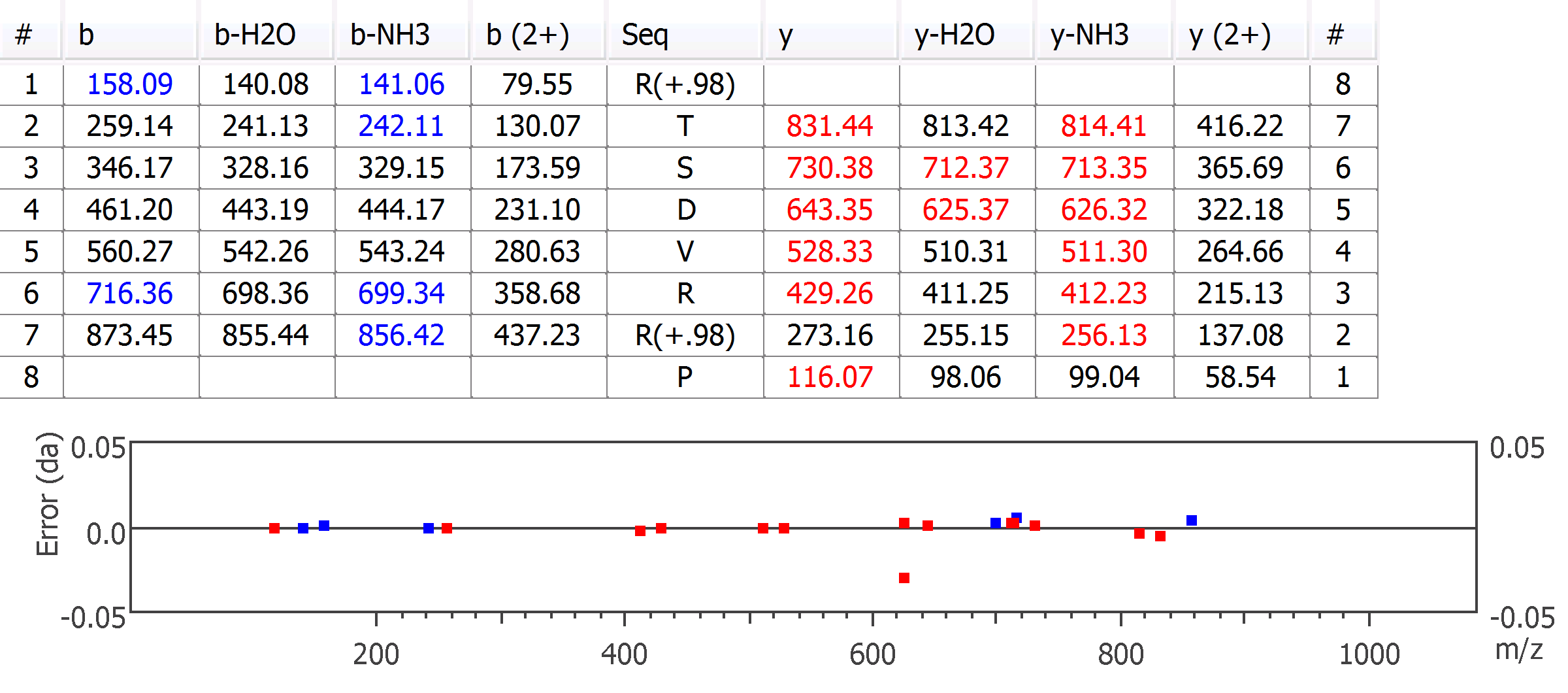


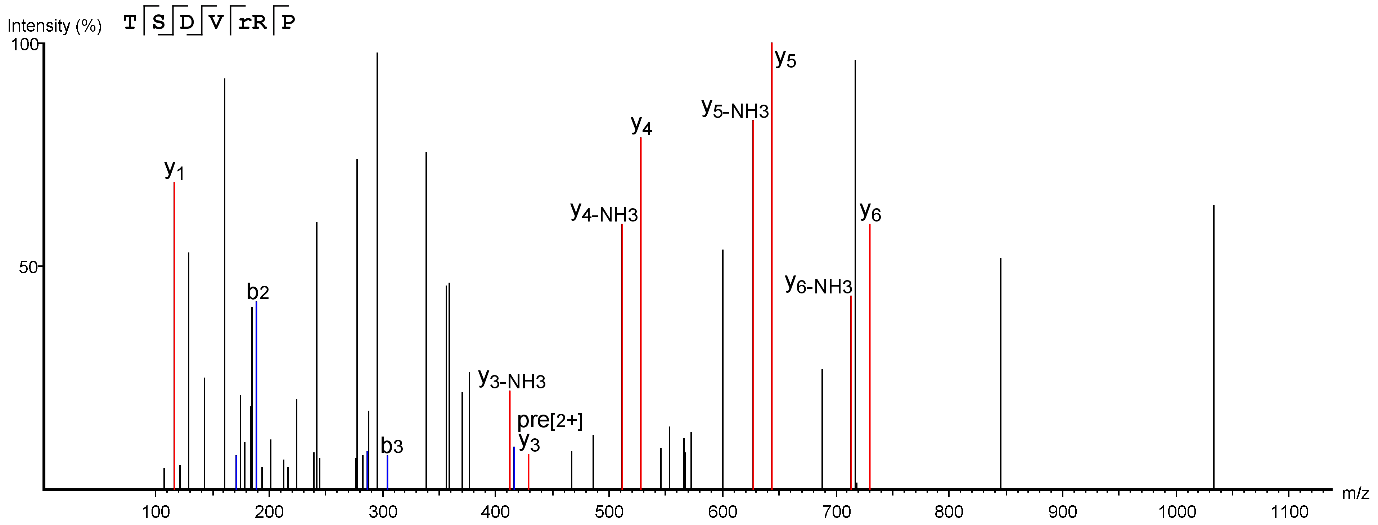

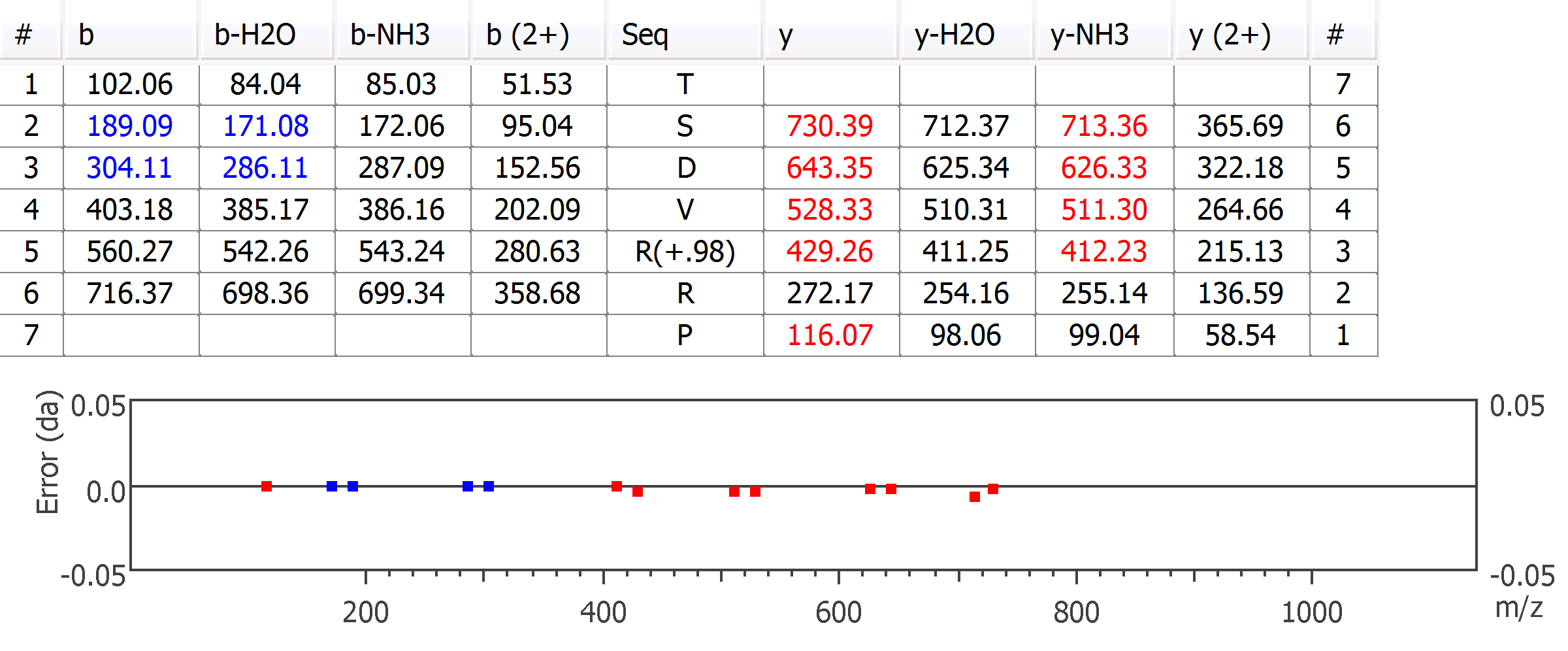

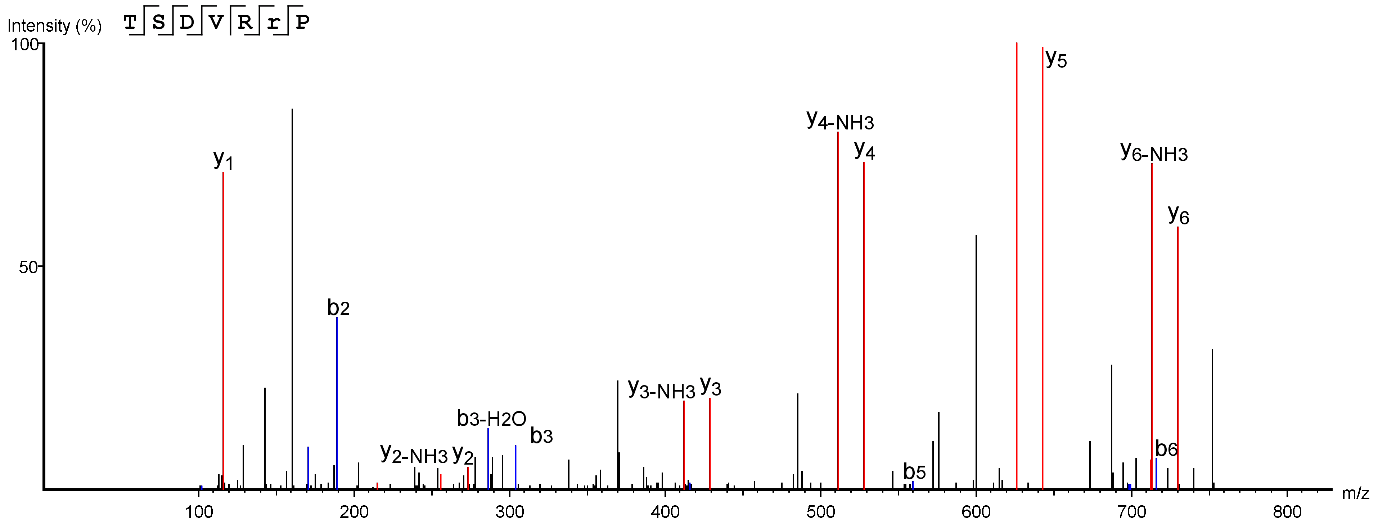

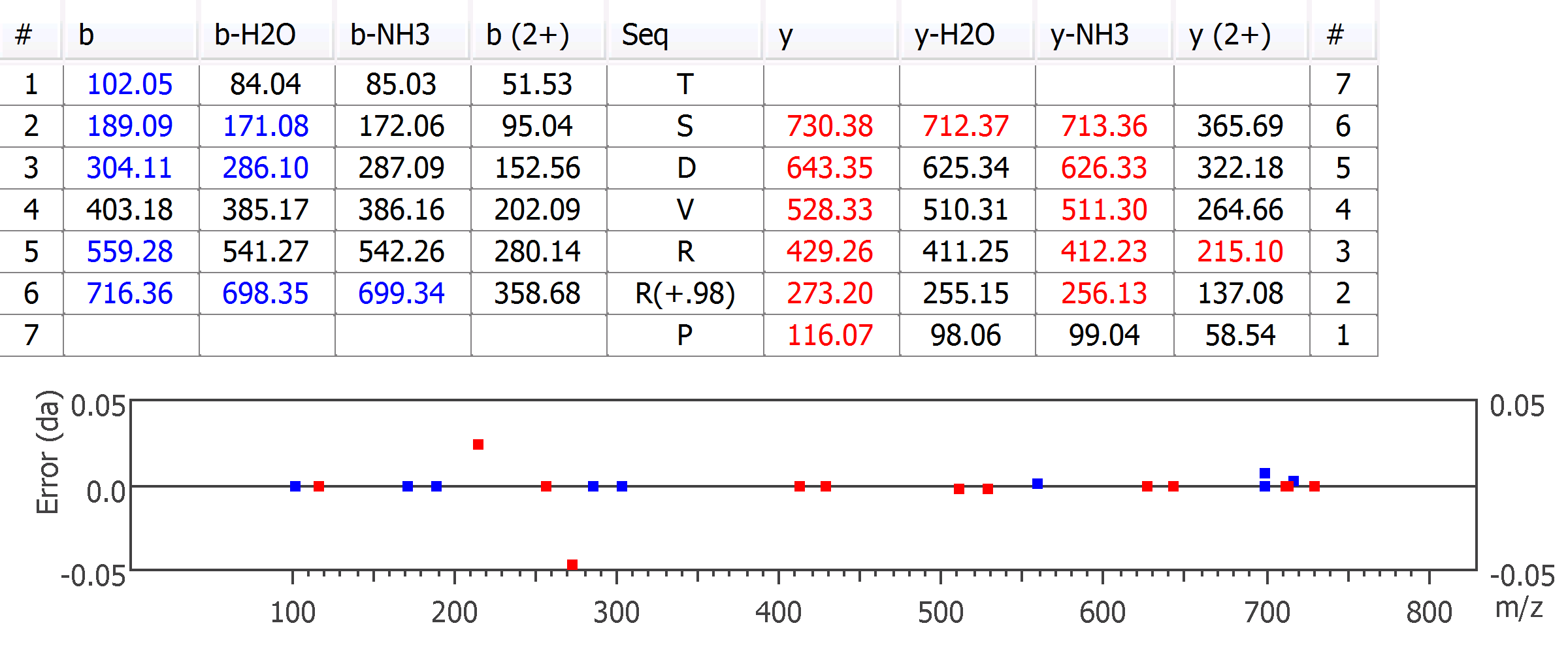


**
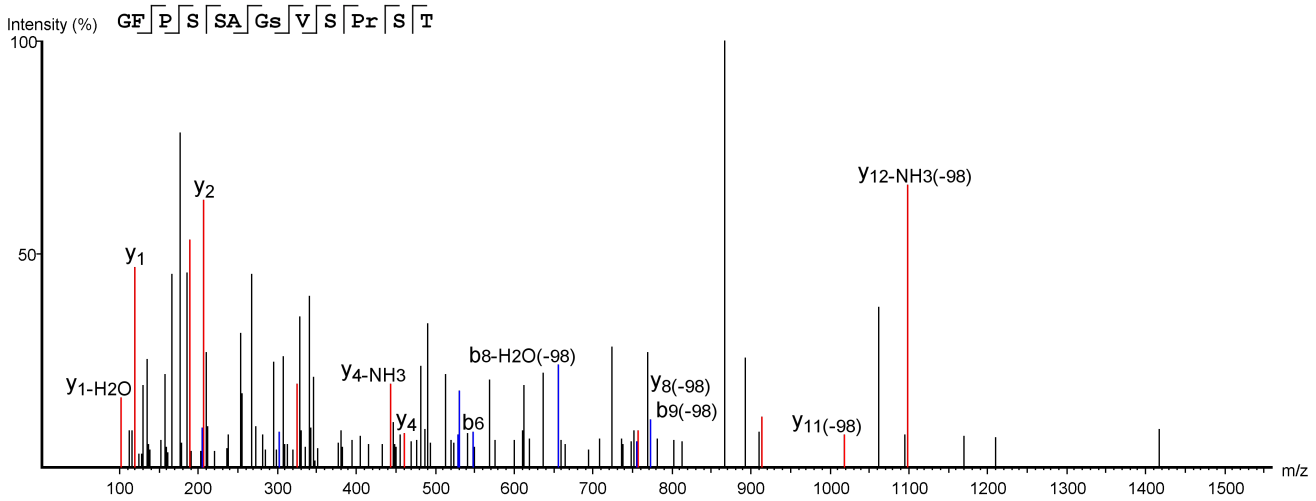

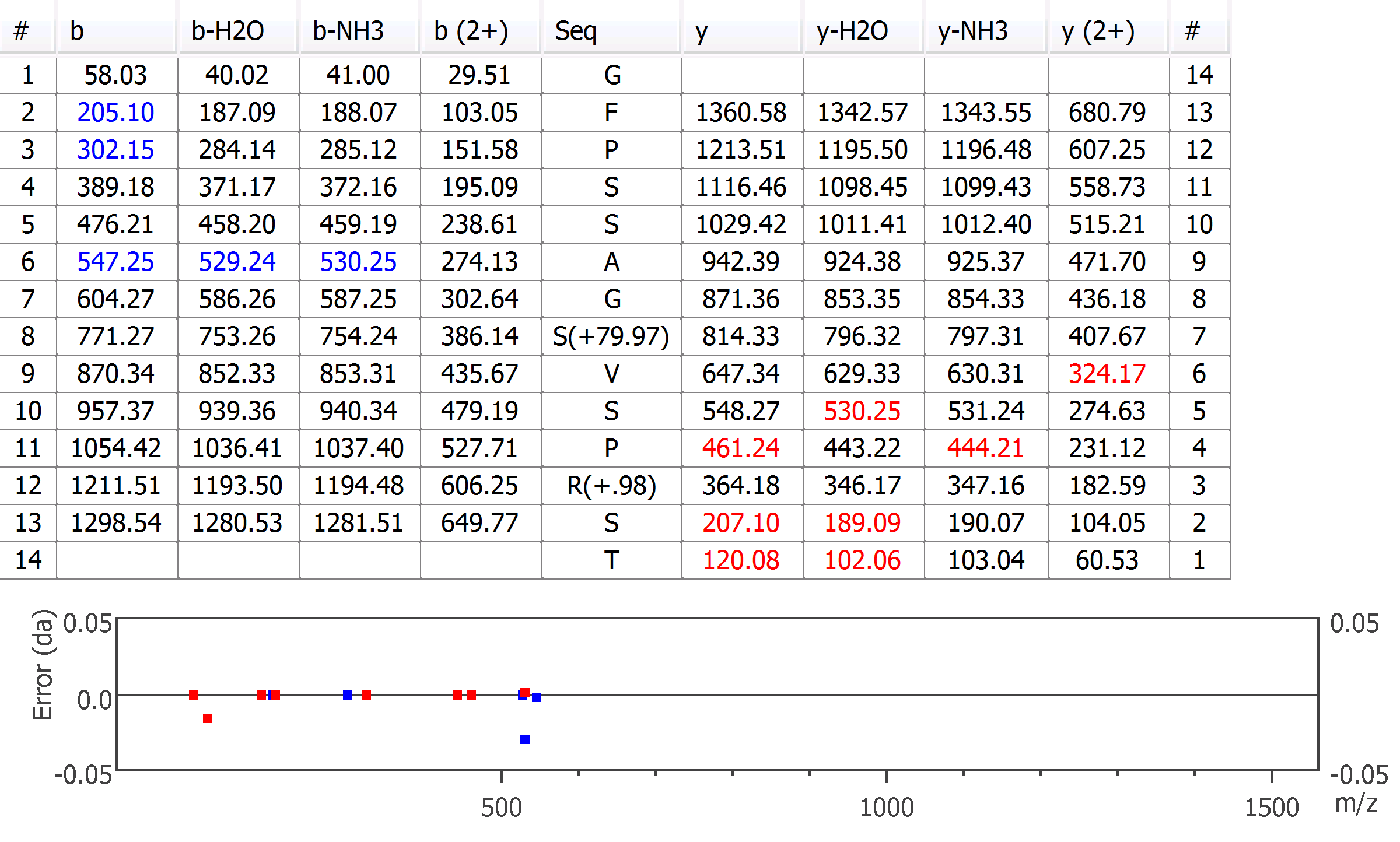
**


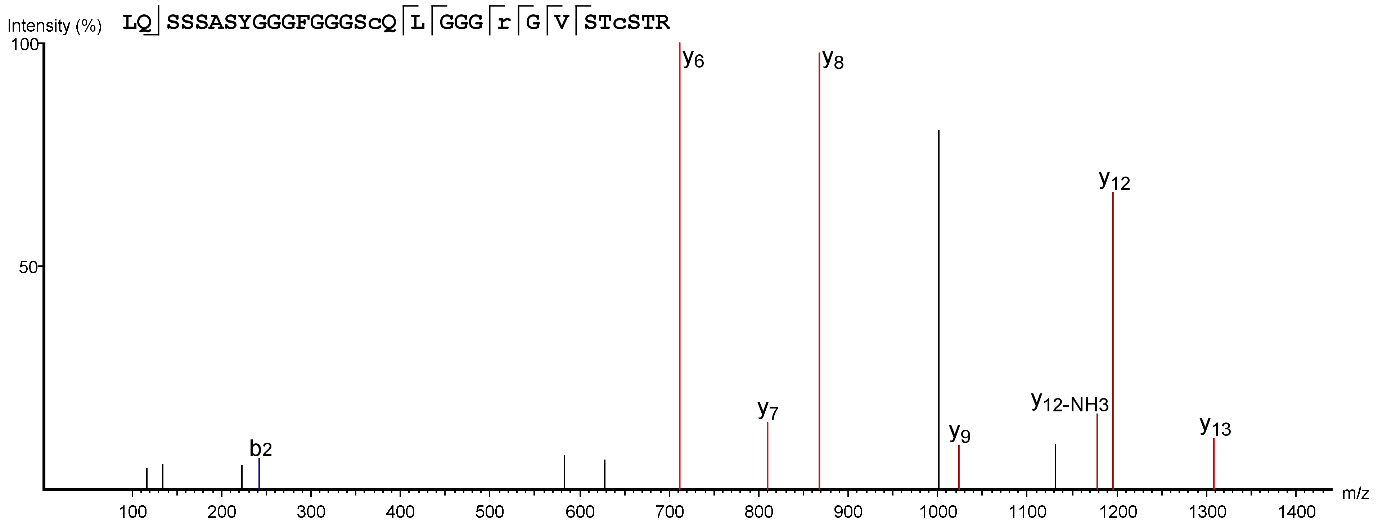

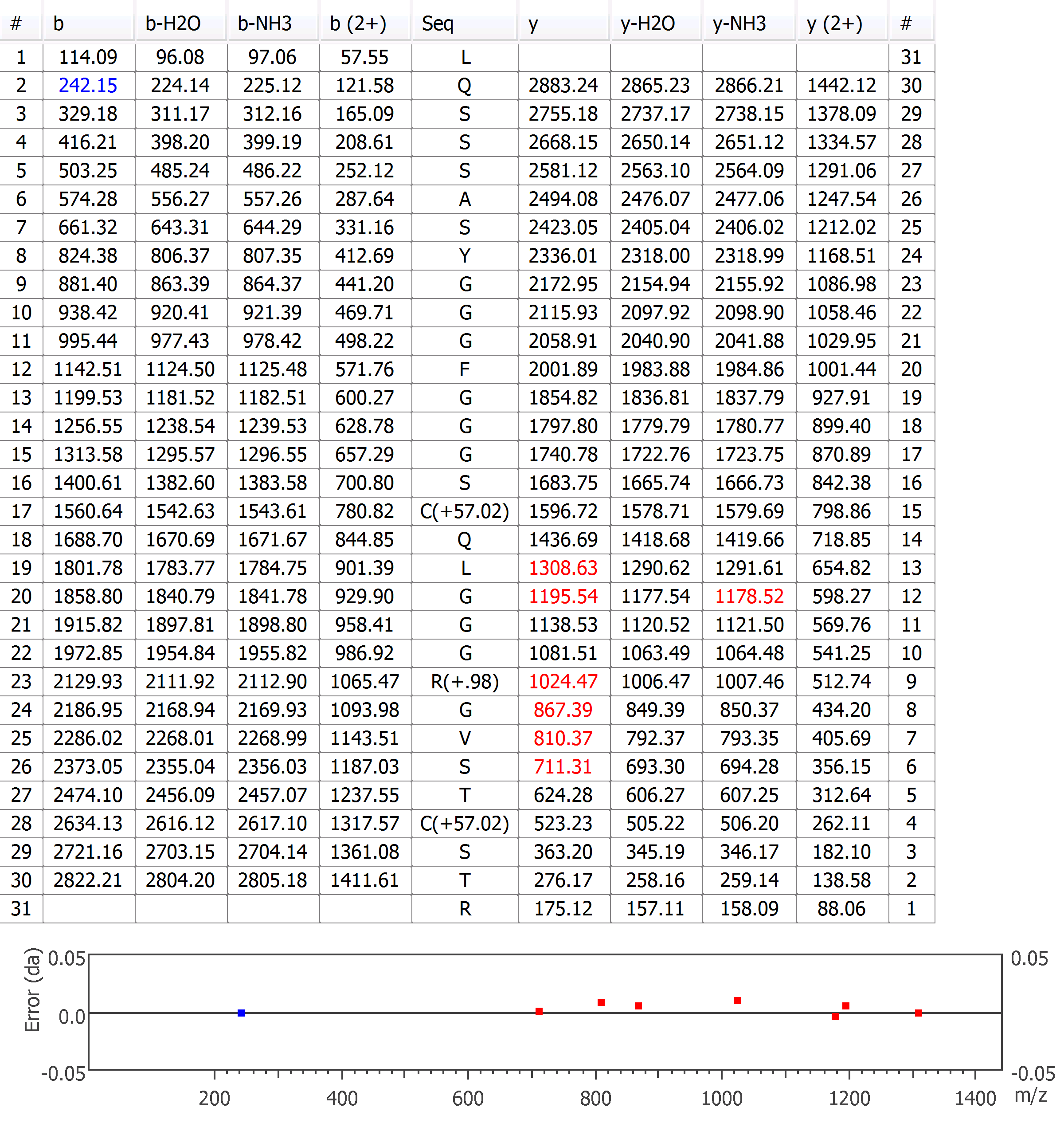


**Figure 2.** ACPA levels in PD positive (+) and negative (-) RA patients. Red lines indicates mean. * p < 0.05, ** p < 0.01, ns – not significant. PD, periodontitis; RA, rheumatoid arthritis; cFIBβ, citrullinated fibrinogen β; cTNC5, citrullinated tenascin-C; CEP-1, citrullinated α-enolase peptide 1; cVIM, citrullinated vimentin; cCK13-1, citrullinated cytokeratin; CCP2, cyclic citrullinated peptides 2.

**Figure 3.** Anti-RgpB levels in A) PD positive (+) and negative (-) patients B) OA and RA patients and C) CCP2 positive (+) and CCP2 negative (-) RA patients. Red lines indicates mean. * p < 0.05, **** p < 0.0001, ns – not significant. RgpB, arginine gingipain B; OA, osteoarthritis; RA, rheumatoid arthritis; CCP2, cyclic citrullinated peptides 2; PD, periodontitis.
